# VlincRNAs controlled by retroviral elements are a hallmark of pluripotency and cancer

**DOI:** 10.1186/gb-2013-14-7-r73

**Published:** 2013-07-22

**Authors:** Georges St Laurent, Dmitry Shtokalo, Biao Dong, Michael R Tackett, Xiaoxuan Fan, Sandra Lazorthes, Estelle Nicolas, Nianli Sang, Timothy J Triche, Timothy A McCaffrey, Weidong Xiao, Philipp Kapranov

**Affiliations:** 1 St. Laurent Institute, One Kendall Square, Cambridge, MA; 2 Department of Molecular Biology, Cell Biology, and Biochemistry, Brown University, Providence, RI; 3 A.P.Ershov Institute of Informatics Systems SB RAS, 6, Acad. Lavrentjev ave., Novosibirsk 630090, Russia; 4 Department of Microbiology and Immunology, Sol Sherry Thrombosis Research Center, Temple University, Philadelphia, PA; 5 Université de Toulouse, UPS, LBCMCP, F-31062 Toulouse, France; 6 CNRS, LBCMCP, F-31062 Toulouse, France; 7 Department of Biology, Drexel University, 3245 Chestnut St, PISB 417, Philadelphia, PA; 8 Department of Pathology, University of Southern California, 1975 Zonal Avenue, Los Angeles, CA; 9 The George Washington University Medical Center, Department of Medicine, Division of Genomic Medicine, 2300 I St. NW, Washington, D.C

**Keywords:** vlincRNA, LTR, macroRNA, cancer, embryonic stem cell, non-coding RNA, intelligent scaffold, single molecule sequencing, RNAseq

## Abstract

**Background:**

The function of the non-coding portion of the human genome remains one of the most important questions of our time. Its vast complexity is exemplified by the recent identification of an unusual and notable component of the transcriptome - very long intergenic non-coding RNAs, termed vlincRNAs.

**Results:**

Here we identify 2,147 vlincRNAs covering 10 percent of our genome. We show they are present not only in cancerous cells, but also in primary cells and normal human tissues, and are controlled by canonical promoters. Furthermore, vlincRNA promoters frequently originate from within endogenous retroviral sequences. Strikingly, the number of vlincRNAs expressed from endogenous retroviral promoters strongly correlates with pluripotency or the degree of malignant transformation. These results suggest a previously unknown connection between the pluripotent state and cancer via retroviral repeat-driven expression of vlincRNAs. Finally, we show that vlincRNAs can be syntenically conserved in humans and mouse and their depletion using RNAi can cause apoptosis in cancerous cells.

**Conclusions:**

These intriguing observations suggest that vlincRNAs could create a framework that combines many existing short ESTs and lincRNAs into a landscape of very long transcripts functioning in the regulation of gene expression in the nucleus. Certain types of vlincRNAs participate at specific stages of normal development and, based on analysis of a limited set of cancerous and primary cell lines, they appear to be co-opted by cancer-associated transcriptional programs. This provides additional understanding of transcriptome regulation during the malignant state, and could lead to additional targets and options for its reversal.

## Background

Over the past 10+ years, efforts to understand the complexity of the human transcriptome have employed a number of independent technologies to discover and characterize the extent of transcription from non-coding regions [1-4]. While little doubt remains about the existence of this "dark matter" RNA, as highlighted by the recent ENCODE papers [5,6], its function and biological significance continues to generate controversy [3,7-11]. Much of the problem stems from an insufficient theoretical basis to classify and categorize the dark matter transcripts, and thus they can appear as transcriptional 'noise' made by the cell. Another part of the problem arises from our genome's large number of repetitive sequences of retroviral origin. The presence of so many of these poorly conserved elements is often mentioned to support the notion that the non-coding portion of the genome has little function.

Recently, by profiling total RNA depleted of ribosomal RNAswith single-molecule sequencing (SMS), we revealed an intriguing phenomenon: the presence of very long (50 to ~700 kb) transcribed regions of human non-coding intergenic genomic space [12]. These regions, dubbed vlincRNAs for very long intergenic non-coding regions, were found in tumors, and showed highly cell-type specific expression patterns. Unlike many non-coding RNAs with low levels of expression, RNAs produced from these novel vlincRNA regions were relatively abundant by mass in the cell [12]. The length of these regions emerged as a prominent feature, which, together with an absence of any obvious coding potential, made them similar to macroRNAs such as Airn or KCNQ1OT1, yet distinct from the previously identified long-intergenic ncRNAs (lincs) [13]. MacroRNAs are very long, un-spliced RNAs driven by canonical RNA Pol II promoters that function in the maintenance of imprinting in normal tissues [14,15]. However, only a few characterized examples of macroRNAs exist, leading to the belief that they occur only rarely in the human genome [14,15].

Here we show that vlincRNAs encode functional RNAs whose combined properties parallel those of the macroRNAs except that they criss-cross our genome in very large numbers. Regulated by relatively few dedicated promoters, these very large transcripts account for much of the dark matter transcription in the cell. Strikingly, we show that retroviral repeats, the quintessential dark matter of the genome, tend to specifically associate with tissue-specific vlincRNAs. This association occurs in both transformed and pluripotent cells, pointing to yet another un-anticipated link between pluripotency and malignancy.

## Results

### VlincRNAs exist in very large numbers in a variety of cancerous and normal cell types

In this work, we identify and characterize over 2000 vlincRNAs found in a number of published and unpublished datasets (Table 1). We generated a Single Molecule Sequencing (SMS) RNA-seq dataset from human blood (Materials and Methods) in order to profile a human primary tissue containing diverse cell types, free of cell culture or other *in-vitro *artifacts. In addition, we used the ENCODE/CSHL long nuclear polyA-RNAseq dataset [5,6] consisting of several cell lines of cancerous or primary origin (Materials and Methods). First, we determined the reproducibility of our original vlincRNA observations [12] by verifying their detection on different sequencing platforms, by different research groups, and in different RNA isolations from the same cell line. Of the 60 vlincRNAs previously identified by us on the Helicos SMS platform as specific to the K562 human cell line [12] (see below), 56 were expressed in the ENCODE K562 long RNAseq dataset generated with the Illumina platform. This result confirmed that vlincRNAs are *bona fide *RNAs present in a cell. Overall, 341 of the original 580 tumor vlincRNAs [12] (Table 1) overlap ENCODE vlincRNAs identified from mostly different cell lines, using a different sequencing platform (p-value 1.54 × 10^-45^).

**Table 1:** Total number of vlincRNAs and overlap with All promoters in different cell lines and tissues

Source	Cell line/Tissue	Cell Type	Number of vlincRNAs	VlincRNAs Overlapping with All Promoters	Overlap Expected by Chance	Fold over chance	p-value
Kapranov et al[12]	K562 + Ewing Sarcomas	Malignant	580	336*	131.7	2.5	2.4E-74

This work	Blood	Normal Tissue	613	321*	142.5	2.2	7.7E-55

ENCODE	K562	Malignant	407	216**	23.2	9.3	1.3E-153

ENCODE	HepG2	Malignant	501	192**	34.1	5.6	7.5E-91

ENCODE	HeLaS3	Malignant	206	N/D	N/D	N/D	N/D

ENCODE	GM12878	Immortalized	591	128**	27.5	4.6	3.6E-48

ENCODE	NHEK	Primary	248	51**	12.1	4.2	2.28 E-18

ENCODE	HUVEC	Primary	340	86**	13.1	6.5	1.9E-44

ENCODE	H1-hEsc	Embryonic Stem	469	205**	24.9	8.2	1.9E-130

This work	Mouse Lung	Normal Tissue	225	115*	49.3	2.3	7.6E-22

* Overlap was done on combined ENCODE All promoter dataset from all 9 cell lines

** Overlap was done on the All ENCODE promoter dataset from the same cell line

VlincRNAs were found in all cell lines tested, including non-cancerous cell lines and tissues (Table 1). Embryonic stem cell line H1-hEsc expressed 469 such regions, while non-cancerous normal human epidermal keratinocyte line (NHEK) and normal human umbilical vein endothelial cells (HUVEC) expressed 248 and 340 vlincRNAs, respectively (Table 1; Additional File 1, Table S1). A complex normal human tissue, blood, revealed expression of 613 vlincRNAs, of which 331 (p-value 2.03 × 10^-49^) overlap ENCODE vlincRNAs. Of 2,285 vlincRNAs found in cancerous or immortalized cells/tissues, 1,088 (47.7%) occurred in the non-cancerous cell lines or blood. While cancerous cells have highly-rearranged genomes and, in theory, could produce non-functional vlincRNA transcripts that reflect those re-arrangements, this argument cannot be made about the primary and embryonic stem cells. This argues that vlincRNAs are not aberrant transcripts resulting from cancerous or tissue-culture conditions. An example of a vlincRNA transcribed in both cancerous as well as embryonic stem cells is shown in the Additional File 2, Figure S1.

The combined number of different non-overlapping vlincRNAs found in K562, Ewing Sarcomas, ENCODE and human blood now totals 2,147, with the longest vlincRNA over 1M bases in length (see Additional File 1, Table S1). Overall, these observations tell us that the human genome encodes thousands of very long regions that give rise to mostly non-coding RNAs that exhibit differential expression not only in cancerous cells, but also in primary cell lines and normal tissues. In total, they cover at least 10% of the genomic space and likely much more, considering that many cell types remain to be profiled.

### VlincRNAs are controlled by normal promoters

**Figure 1:**
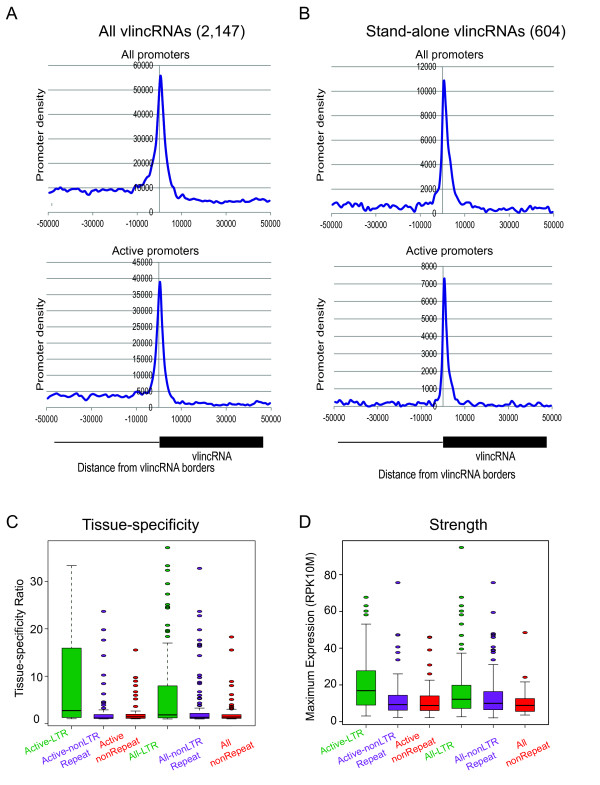
**Properties of vlincRNA-associated promoters**. VlincRNAs tend to have promoters at one of their bounds (A, B) belonging to either the Active or All category of promoters. This tendency was common to all vlincRNAs (A) or the stand alone vlincRNAs (B) defined based on being at least 50 kb away from the nearest gene on either side. Panels A and B show the density of ENCODE promoter coverage (Y-axis) in all 9 ENCODE cell lines (see text) summed up in 1 kb bins +/-50 kb around boundary of each vlincRNA of the 2,147 vlincRNA (see Additional File 1, Table S1). The coordinates on the X-axis refer to middle position of each bin. VlincRNAs assigned to LTR-containing promoters from any of the 9 cell lines tend to be more tissue specific (C) and have a higher RNAseq signal (D).

To explore whether the promoters regulating vlincRNAs have typical chromatin hallmarks, we utilized promoters annotated by the ENCODE consortium in 9 cell lines, consisting of two cancerous lines, an immortalized line, an embryonic stem cell and four primary cell lines [5,16,17] (Materials and Methods). The promoters segregated into 3 categories: "Active", "Weak" and "Inactive/poised", based on the presence of different chromatin marks [16,17]. A clear enrichment of promoters from the three above mentioned classes together ("All" promoter class), and the "Active" class by itself, occurred around the boundaries of the 2,147 vlincRNA regions (Figure 1A). Of the latter, 1,032 (48.06%) and 700 (32.6%) could be associated with All and Active ENCODE promoters, respectively (p-values of 2.82 × 10^-210 ^and 1.0 × 10^-300^) (see Table 1 and Materials and Methods for details).

**Figure 2:**
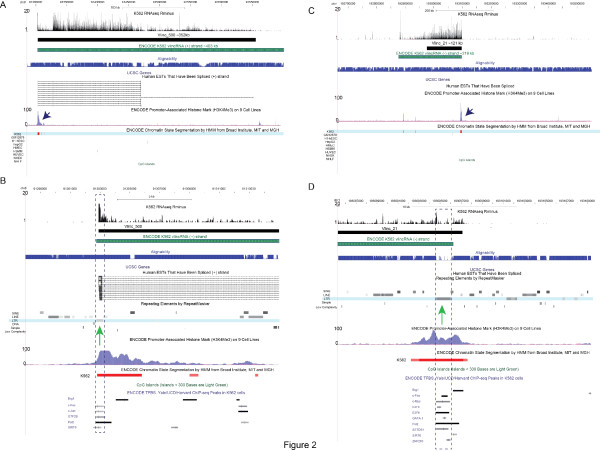
**Examples of two stand-alone K562-specific vlincRNAs that are distal from annotations**. VlincRNAs supported by EST evidence (A & B) and with no EST evidence (C & D) are shown. Zoom-in on the corresponding promoter regions marked by arrows in A& C are shown in the panels B & D. The original 580 vlincRNAs[12] (black, non-strand specific) and the ENCODE vlincRNAs (green, strand-specific) are shown. The vlincRNA designations (vlinc_500 and vlinc_21) refer to Supplementary Table S3 of Kapranov et al [12]. Vlinc_500 (A & B) represents an example of a novel region of ~350 kb whose left bound corresponds fairly well to a cluster of spliced ESTs that share the same 5' ends. Consistent with the K562-specific nature of this region, 9 out of 13 of these ESTs have been isolated from a chronic myelogenous leukemia (CML), the same cancer type as K562, with one of the 9 ESTs spanning almost the entire length of vlincRNA (A). The common 5' end of these ESTs falls within the annotated K562-specific promoter that has within its core an LTR sequence (B). Most of the RNAseq signal was in the intronic regions of these ESTs, consistent with the overall observation that majority of the "dark matter" RNA signal is intronic [12]. Unlike vlinc_500 which is supported by the EST evidence, Vlinc_21 (~121 kb; panels C & D) is located within a totally un-annotated region of chromosome 1 with no spliced EST, ~1.4 Mb from the closest UCSC gene annotation. It also has an LTR repeat at its core (D). RNAseq data shows density of informative reads normalized by 10M informative reads. The Y-axis of the alignability track (Materials and Methods) is on the scale of 0 to 1. Coordinates: hg18.

**Figure 3:**
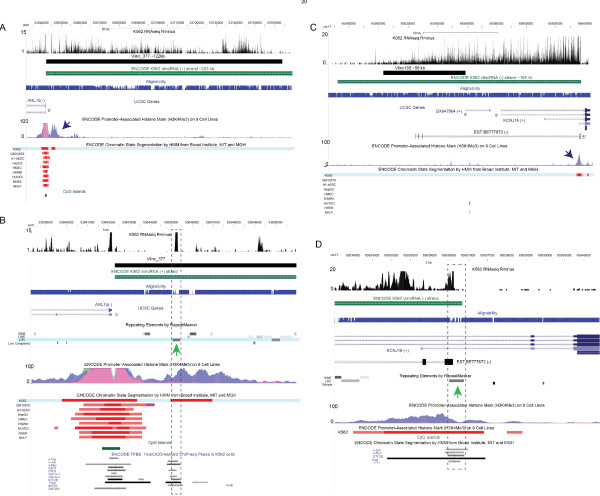
**Examples of two stand-alone K562-specific vlincRNAs that are adjacent (A & B) or overlap (C & D) annotations**. Panels A & B show a vlincRNA potentially produced by bi-directional transcription from a promoter upstream of a known gene. Panels C & D show a vlincRNA antisense to known genes. Zoom-in on the corresponding promoter regions marked by arrows in A& C are shown in the panels B & D. The original 580 vlincRNAs[12] (black, non-strand specific) and the ENCODE vlincRNAs (green, strand-specific) are shown. The vlincRNA designations (vlinc_377 and vlinc_185) refer to Supplementary Table S3 of Kapranov et al[12]. Vlinc_377 (~122 kb, panels A & B) is adjacent to an annotated gene, ARL15. However, it does not appear to be connected to ARL15, but rather represent a stand-alone independently regulated unit. Expression of vlinc_377 was restricted to K562, while ARL15 was constitutively expressed and the direction of vlinc_377 transcription was opposite from ARL15 as shown by the vlincRNA from the ENCODE/CSHL long RNAseq dataset [56] produced with a protocol that reduced spurious second strand formation [57](Materials and Methods). Consistent with this, a K562-specific promoter element could be identified at the boundary of vlincRNA_377, upstream of the constitutive promoter controlling the ARL15 expression. Vlinc_185 (C & D) represents an interesting example of a very long antisense transcript. Due to the original constraint of annotating vlincRNAs as intergenic regions, the vlincRNA bounds did not extend over the KCNJ16 gene even though the RNAseq signal clearly extends into that gene (C). Furthermore, a spliced EST BE777672 connects vlinc_185 to an intron of KCNJ16 and the 5' end of this EST corresponds quite well with the end of the RNAseq signal and also with a K562-specific promoter (C & D). Consistent with this, BE777672 was also isolated from a CML source. The entire length of this vlincRNA would be ~130 kb. Finally, the ENCODE K562 vlincRNA shows the recapitulates the strand and the start site of the previously published vlincRNA. RNAseq data shows density of informative reads normalized by 10M informative reads. The Y-axis of the alignability track (Materials and Methods) is on the scale of 0 to 1. Coordinates: hg18.

VlincRNAs segregate into three categories: totally standalone transcripts (Figure 2), adjacent to known genes, but transcribed bi-directionally (Figure 3 A&B), or antisense to known genes (Figure 3 C&D). Sometimes vlincRNAs correspond to spliced ESTs, but most of the RNAseq signal maps to intronic portions of these ESTs, indicating they probably exist in unspliced form (Figure 2A, Figure 3C). To estimate the fraction of antisense vlincRNAs and those transcribed bi-directionally to known genes, we examined the 1,661 ENCODE vlincRNAs in our dataset with unambiguous strand assignments (Table 1). Of those, 550 (33.1%) were antisense to known genes, and 83 (5.0%) were transcribed bi-directionally. The relatively high fraction of antisense vlincRNAs was reminiscent of estimates of 50-70+% of global antisense transcription obtained in previous genome-wide surveys [18,19]. To exclude the possibility that promoter enrichment comes from nearby human genes, we created a group of 604 stand-alone vlincRNAs from the 2,147 vlincRNAs whose boundaries were more than 50 kb from 5' and 3' boundaries of annotated genes. Very similar to the entire population of vlincRNAs, we observed an enrichment of the ENCODE promoters around the boundaries of such standalone vlincRNAs (Figure 1B): 133 (22.0%) and 222 (36.7%) of the 604 stand-alone vlincRNAs associated with either the Active promoters or All promoters respectively. Again, this was highly significant with p-values of 1.85 × 10^-97 ^and 4.61 × 10^-76 ^.

In theory, poor alignability of short RNAseq reads due to the presence of repetitive elements could affect the 5' boundaries of our vlincRNAs. Using the alignability track generated by the ENCODE project (Materials and Methods), we generated a profile of alignability around the +/- 10 kb window of vlincRNA ends (5' ends for stranded vlincRNAs and both ends for vlincRNAs with no strand). As shown in Additional File 2, Figure S2, reduced alignability occurred in the region 5 kb upstream of the 5' end for 219 out of 2147 vlincRNAs, which could indeed cause some of them to terminate prematurely. However, some vlincRNAs are driven by promoters within repeats (see below) and repeats tend to coincide with regions of poor alignability. Therefore, the RNAseq inferred boundaries adjacent to regions of poor alignability for some of these 219 vlincRNAs could still result from real transcription initiation. Finally, we can estimate that 1,928/2,147 vlincRNAs (~90%) do not suffer from this issue.

Finally, we tested for the presence of long RNAs in two standalone vlincRNA regions by performing a set of overlapping long-range RTPCR reactions (Materials and Methods). As shown by one such example of a standalone, LTR-driven vlincRNA (see below) in Additional File 2, Figure S3, the long-range RTPCR reactions can indeed detect the presence of such RNAs. As expected, no amplification occurred once amplicons extended beyond the RNAseq signal in that cell line even though the same primer set worked well on genomic DNA (see Additional File 2, Figure S3).

### Endogenous retrovirus based promoters frequently drive tissue-specific vlincRNAs

Cell-type specificity constitutes one of the intriguing properties of vlincRNAs, to the point where vlincRNA expression patterns can distinguish different patients with the same tumor type [12]. To investigate this property, we analyzed the promoters of cell-type specific vlincRNAs in K562, since it has annotated promoters, ENCODE RNAseq, and SMS RNAseq data. We identified 53 K562-specific vlincRNAs and noticed a striking enrichment for viral long terminal repeat (LTR) elements in their promoters: 62.9% of active promoters associated with K562-specific vlincRNAs overlapped or fully contained an LTR repeat sequence (see Additional File 2, Figure S4)

Repeats in general are known to drive cell type-specific expression [20], as the K562 promoter landscape demonstrates: 26.7% of K562 specific active promoters overlapped LTRs, notably higher than that of all Active promoters (7.7%) (Table 2). However, as mentioned above, the vlincRNA associated fraction was far higher at 62.9%. For comparison, the same fraction associated with K562-specific known genes was only 25.4% (see Additional File 2, Figure S4). Reciprocally, 75.6% of the K562-specific vlincRNAs could be assigned to at least one K562-specific Active promoter with an LTR. The corresponding fraction for K562-specific Known Genes was ~3x lower, 26.3%. Thus, LTR-containing promoters strongly associate with tissue-specific vlincRNAs, more so than all tissue-specific Active promoters or tissue-specific transcripts encoding proteins (see Additional File 2, Figure S4).

**Table 2:** Distribution of LTRs in Active promoters in malignant, immortalized, primary and embryonic stem cells.

	Cell Type	Total Promoters	Total Promoters that overlap LTRs	% of Total Promoters that overlap LTRs	Cell-line Specific Promoters	% Cell-line Specific Promoters	Cell-line Specific Promoters that overlap LTRs	% of Cell line Specific Promoters that overlap LTRs	Number of LTRs that overlaps cell-line specific Promoters
K562	Malignant	15,627	1,199	7.7%	1,599	10.2%	427	26.7%	622

HepG2	Malignant	15,993	1,165	7.3%	1,886	11.8%	303	16.1%	400

GM12878	Immortalized	15,279	989	6.5%	1,613	10.6%	153	9.5%	203

NHLF	Primary	14,888	546	3.7%	259	1.7%	15	5.8%	16

HMEC	Primary	13,711	535	3.9%	145	1.1%	9	6.2%	12

HSMM	Primary	14,335	537	3.7%	305	2.1%	23	7.5%	25

HUVEC	Primary	12,319	397	3.2%	102	0.8%	6	5.9%	7

NHEK	Primary	14,009	700	5.0%	219	1.6%	24	11.0%	29

H1-hESC	Embryonic Stem	12,477	568	4.6%	453	3.6%	217	47.9%	308

**Figure 4:**
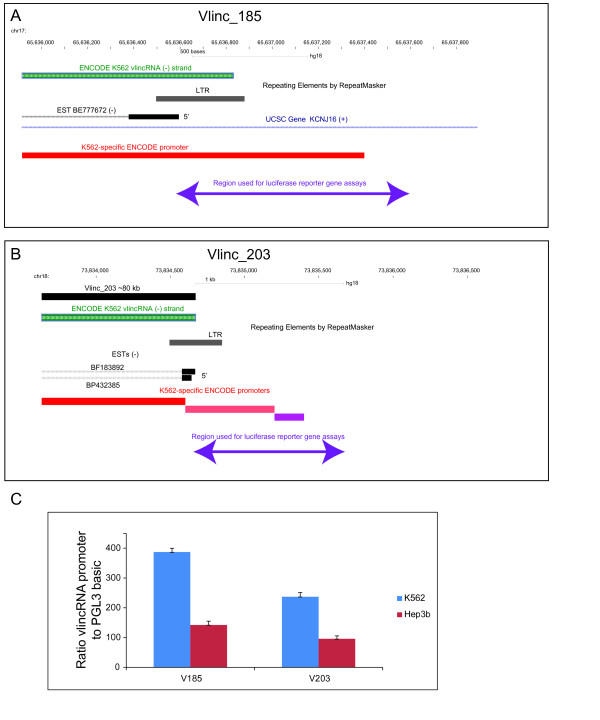
**Luciferase reporter assays**. Diagrams of the regions used for the promoter assays (A & B) and the actual results (C) (Materials and Methods) are shown. The original 580 vlincRNAs (black, non-strand specific) and the ENCODE vlincRNAs (green, strand-specific) are shown (A&B). Also shown are the positions of the LTR repeats as annotated by the RepeatMasker, ENCODE K562 promoters (see text), ESTs and the actual regions used for the reporter gene assays (A &B). Error bars show standard deviations of different biological replicas.

To further validate the contribution of LTRs in vlincRNA promoters, we used the ENCODE Transcription Factor Binding Sites [5,21] (Materials and Methods). Overall, 88.2% of the vlincRNA LTR promoters bind RNA pol II and other transcription factors directly to the LTR sequences, as exemplified in Figures 2 and 3. Notably, the K562-specific vlincRNAs and the associated LTR-containing promoters were found by two very different means (RNAseq and ChIP-seq) performed by two different groups on totally different batches of K562 cells. This consistency argues for a tight control over cell type-specific activation of these LTR-based promoters and against spurious transcriptional noise. To show that LTR-containing sequences from Active promoters associated with vlincRNAs can indeed function to initiate transcription, we selected 2 LTR-containing K562-specific Active promoters and performed reporter gene assays in two cell lines (K562 and Hep3b) (Figure 4, Materials and Methods). The sequences used for the assays contained 700-800 bp of upstream genomic flanking regions in addition to 200-300 bp of LTR (Figure 4, Materials and Methods). As expected, the sequences functioned as strong promoter elements, driving expression 200-400 fold above the empty vector in K562 (Figure 4). They were also active in Hep3b, albeit at lower levels than in K562 (Figure 4), suggesting that additional elements and/or trans-acting factors regulate specific activation of these sequences in K562 and repression in other cell lines (see below). Endogenous LTRs are known to harbor promoter and enhancer elements, and a number of well characterized LTR-based promoters of known genes exist[22]. However, to our knowledge, this is the first description of an association between LTRs and a very large percentage of the genome that produces non-coding RNA species with the exception of a recent paper by Kelley and Rinn that showed association of LTR repeats with a different class of non-coding RNAs (see below) [23].

Interestingly, only LTR-type repeats appear to confer strong tissue-specific expression patterns on vlincRNAs. To examine this, we used the original 580 vlincRNAs for which we had highly quantitative SMS RNAseq data from total cellular RNA (Table 1). We split the Active and All promoters according to whether they contained an LTR, did not contain an LTR but contained another retroposon, or contained no retroposon repeats, and categorized the 580 vlincRNAs based on their promoter association into one of the three above classes. We defined vlincRNA tissue-specificity, for any tissue in our previous SMS RNAseq dataset [12], as the ratio of the highest expresser to the second highest expresser. The vlincRNAs associated with LTR promoters showed much higher tissue-specificity than either the non-repeat or the non-LTR repeat-containing promoters (Figure 1C). In addition, the vlincRNAs associated with LTR promoters had higher expression levels than vlincRNAs associated with other promoters (Figure 1D).

### Association between vlincRNAs and retroviral promoters is a function of transformed or pluripotent state in key cell lines and primary cells

Using the ENCODE promoter data with normal cells as well as cell lines at different levels of transformation, we observed a direct relationship between the number of Active promoters that overlapped LTRs and the replicative state of the cell. The primary cell lines had the lowest number of such promoters (397-700), followed by the immortalized cell line (989), and finally the cancerous cell lines (1,165-1,199) as shown in Table 2. For each cell line, we then determined a set of cell-line specific Active promoters by removing any Active promoter that overlapped Active, Weak or Inactive/poised promoters in the other 8 cell lines. The same trend held true for the cell line-specific Active promoters: the respective numbers were: 6-24, 153 and 303-427 (Table 2). However, the embryonic stem cell line H1-hEsc stood out in this category from the other primary cell lines. With 217 specific Active promoters, H1-hEsc had the highest fraction of specific Active promoters that overlapped LTRs compared to all other cell lines - 50% (Table 2). This was followed by the cancerous cell lines (16.1%-26.7%), the immortalized cell line (9.7%) and finally the primary cell lines with an average of 7.3% (highly variable, most likely due to the low number of promoters that overlapped LTRs) (Table 2). In summary, while a few LTR's were found in Active promoters (both cell-line specific and not) of normal primary cells, far higher numbers were found in Active promoters of stem cells and cancerous cells.

We have presented evidence that vlincRNAs associate with LTR promoters and that malignant and pluripotent cells recruit LTR promoters much more frequently than primary cells. We next wanted to close the logical loop and determine whether cancerous and pluripotent cells had a higher tendency to express LTR-driven vlincRNAs. We compared vlincRNAs and annotated promoters from 6 cell lines common to the ENCODE/CSHL long RNAseq dataset and ENCODE annotated promoters (Materials and Methods). The malignant/pluripotent cell lines indeed expressed an order of magnitude higher absolute number and fraction of vlincRNAs driven by the LTR promoters than the primary cell lines (Table 3). The embryonic stem cell line expressed similar numbers to the cancerous cell lines, while the immortalized cell lines expressed numbers in between the ES cells and the primary cells (Table 3).

**Table 3:** Enrichment of LTR-driven vlincRNAs in malignant, immortalized, primary and embryonic stem cells.

Cell line/Tissue	Cell Type	Number of vlincRNAs	VlincRNAs Assigned to LTR* promoters	% Assigned to LTR promoters	Overlap Expected by Chance	Fold over chance	p-value
K562	Malignant	407	119	29.23%	4.5	26.6	7.2E-130

HepG2	Malignant	501	71	14.17%	6.3	11.4	7.9E-51

GM12878	Immortalized	591	47	7.95%	3.5	13.5	7.1E-37

NHEK	Primary	248	1	0.40%	1.1	0.9	0.67

HUVEC	Primary	340	10	2.94%	1.0	10.0	9.4E-08

H1-hEsc	Embryonic Stem	469	68	14.49%	3.5	19.6	5.4E-64

*Defined as All promoters found in the respective cell line and overlapping an LTR

To validate this intriguing observation in a larger number of cell types, we investigated expression of vlincRNAs in 16 cell lines using ENCODE total polyA-RNAseq data: the 6 ENCODE cell lines in Table 3 and 10 additional cell lines, including 3 cancerous and 7 primary [24] (Materials and Methods). In this analysis we compared vlincRNAs that could be assigned to LTR-containing promoters vs vlincRNAs assigned to promoters with no LTRs. The ratio expression of each vlincRNA in cancerous, immortalized or ES cells compared to the primary cells was calculated (Materials and Methods). The averages of these ratios for LTR and nonLTR vlincRNAs are shown in Table 4 (Materials and Methods). Both, LTR and nonLTR vlincRNAs showed much higher expression in malignant, immortalized or ESC cells than in primary cells (Table 4). However, as expected, the upregulation in cancerous, immortalized and ES cells for the LTR-vlincRNAs was consistently ~10 fold higher compared to the nonLTR vlincRNAs (Table 4). The cancerous and ESC lines had similarly high levels of upregulation relative to primary cells, while the immortalized cells showed less (Table 4). This result is again consistent with LTR-driven vlincRNAs being more highly expressed and more specific to malignant and pluripotent cell types.

**Table 4:** Upregulation of vlincRNAs in malignant, immortalized and embryonic stem cell lines.

	LTR vlincRNAs	non-LTR vlincRNAs
Max* Cancer vs Max Primary	921.2	123.9

Immortalized vs Max Primary	271.5	20.8

ESC vs Max Primary	1,094.4	102.3

*Maximum level in the cancerous or primary cell lines.

### Different cell types exhibit diverse patterns of different types of LTRs activated as vlincRNA promoters

Overall, we detect 8 different types of retroviral elements enriched in promoters of vlincRNAs in the 6 ENCODE cell lines (Table 5, Materials and Methods). The primary cell lines NHEK, HUVEC, and immortalized GM12878 exhibited no significant enrichment of any particular type of LTR and thus are not shown. We tested enrichment of retroviral elements present on the sense strand only, and on either strand, relative to the strand of the corresponding vlincRNAs. In the embryonic stem cells we observed enrichment of HERVH-int and LTR7 in vlincRNA promoters (Table 5), similar to that previously observed for lincRNA promoters [23]. However, unexpectedly we also saw a wide degree of variation between cell types, with each cell type having a unique pattern of LTR-elements (Table 5). For example, while the most enriched element in K562 was LTR12C, it was not enriched in any of the other 5 cell lines. Interestingly, LTR12 was previously shown to serve as enhancer and drive expression of long non-coding RNAs in the beta-globin locus also in K562 cells [25,26]. Similarly, HERVH-int was enriched in H1-Esc and in K562, but not in any other cell line. Reciprocally, MER1-int, Harlequin-int and HERVE_a-int were enriched in vlincRNA promoters in HepG2 cell line, but not in any other (Table 5).

**Table 5:** Enrichment of different LTR elements^1 ^in vlincRNA promoters

K562			HepG2			H1-Esc		
**Repeat**	**# repeats^2^**	**Fold^4 ^(p-value)**	**Repeat**	**# repeats^2^**	**Fold^4 ^(p-value)**	**Repeat**	**# repeats^2^**	**Fold^4 ^(p-value)**

	Both Strands^**3**^							

LTR12C	30	9.48 (1.1E-20)	MER61-int	4	22.62 (2.5E-05)	HERVH-int	46	9.49 (7.9E-30)

LTR1	8	8.23 (4.6E-06)	Harlequin-int	4	9.37 (8.8E-04)	LTR7	46	21.66 (1.42E-46)

THE1A	7	10.49 (3.2E-06)						

	Sense Strand							

LTR12C	15	7.48 (2.4E-09)	MER61-int	4	39.50 (3.7E-06)	HERVH-int	20	5.98 (3.4E-09)

LTR1	7	12.71 (1.2E-06)	Harlequin-int	4	14.96 (2.1E-04)	LTR7	20	14.02 (5.4E-17)

THE1A	6	16.61 (1.4E-06)	HERVE_a-int	2	48.54 (9.2E-04)			

^1^Out of 505 different types of LTR elements listed in RepeatMasker

^2^Number of the collapsed retroviral elements (see Materials and Methods) in promoters of vlincRNAs

^3^Non-strand specific analysis. Repeats in both sense and antisense direction relative to the strand of the associated vlincRNAs were counted.

^4^Fold enrichment of what would be expected by random and the corresponding p-value. Elements with the minimum p-value of 10^-4^, are given in the ranking order. To obtain p-value after Bonferroni correction multiple by 505 in non-strand specific analysis or 201 in the sense strand analysis.

The "-int" elements denote the internal portions of complete proviruses composed of "-int" regions flanked by LTRs. While this analysis showed enrichment of only the internal portions in HepG2, the majority vlincRNA promoters that mapped to the "-int" regions also overlapped adjacent LTRs. Of the 16 HepG2 vlincRNAs whose promoters mapped to complete proviruses, 15 also overlapped LTRs. While it is possible that the internal regions of complete proviruses may also contain promoters, at present we cannot distinguish whether the initiation happens in LTRs only or in the internal regions. Thus, enrichment of the '-int" element in this study primarily refers to enrichment of the corresponding complete proviruses including their LTRs.

Some ERV elements like Harlequin-int were enriched only in the sense polarity to the corresponding vlincRNAs. Others like HERVH-int in H1-Esc were enriched equally on the sense or antisense strand relative to the vlincRNAs (Table 5). The latter is likely at least in part due to bi-directional transcription that we sometimes observed from complete proviruses that have both functional LTRs promoters (data not shown). In addition, LTRs are known to contain bi-directional promoters [27,28].

### VlincRNAs represent functional RNAs

There are many ways to determine the functional importance of a class of RNAs. Syntenic conservation between species often provides evidence of function, especially in non-coding RNA with low sequence conservation [29]. To investigate the presence of vlincRNAs in the same syntenic locations in human and mice, we generated a vlincRNA dataset from normal mouse lung (Materials and Methods). In total, 225 mouse vlincRNAs were found and mapped to the human genome in intergenic space (Table 1). Similar to the human vlincRNAs, the mouse vlincRNAs overlap the ENCODE promoters (Table 1) and in addition, 79/225 mouse vlincRNAs overlapped human vlincRNAs (p-value 0.03). This argues for conservation, and thus, functional importance of this class of RNAs. In addition to synteny, comparisons to other independent datasets of known functional importance can be informative. Analyses show that human vlincRNAs overlap disease-associated human SNPs found in multiple GWAS studies (Materials and Methods) to an extent much greater than that expected by chance: 372/2,147 vlincRNAs overlapped 576 such SNPs (p-value 0.0134).

**Figure 5:**
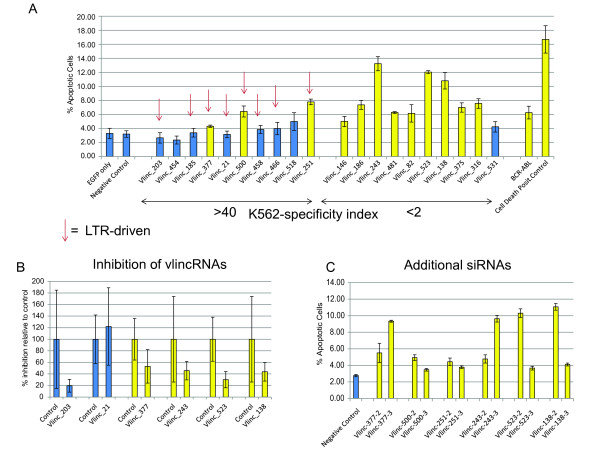
**Results of vlincRNA RNAi inhibition study**. Percent of apoptotic GFP-positive cells (Y-axis) is shown for each indicated treatment (X-axis) for original set of 20 vlincRNAs with 1 siRNA per vlincRNA (A) and for the validation set where 2 additional independent siRNAs per vlincRNA were designed (C). Depletion of selected vlincRNAs was confirmed using real-time RT-PCR (B). "EGFP only" is transfection with EGFP-expressing plasmid only, all other designations imply co-transfection of EGFP-expressing plasmid with the indicated siRNA. Treatments highlighted in yellow are significant at p-value of < 0.05 (Student one-sided t-test). Error bars show standard deviations of 3 independent transfections (A, C) or RT-PCR reaction (B). Results from the negative control siRNA, positive control siRNA against BCR-ABL and AllStars Hs Cell Death positive control siRNA blend are also shown. The vlincRNA data is sorted by the K562-specificity index (see text) with the most K562-specific vlincRNAs on the left (A). Additional details in text and Materials and Methods. The original data is shown in Additional File 3, Table S3.

Finally, to directly ascertain whether vlincRNAs could impact cell survival, we conducted siRNA knockdown experiments with 20 vlincRNAs, 10 K526-specific and 10 found in K562 and other cell lines, and assayed cells for apoptosis (Materials and Methods). We co-transfected a plasmid expressing EGFP reporter and assayed % apoptotic cells in GFP-positive K562 cells using flow cytometry (Figure 5A; Additional File 3, Table S3; Additional File 2, Figures S5-S9). Overall, treatment with siRNAs against 12/20 vlincRNAs resulted in a percentage of apoptotic cells significantly higher than in the negative siRNA control (p-value <0.05) (Figure 5A, Additional File 3, Table S3). siRNA treatments against 9 out of the 12 positive vlincRNAs resulted in the same or higher effect than that of siRNA to a known cancer fusion gene, BCR-ABL (Figure 5A, Additional File 3, Table S3). Notably, the results also revealed a trend where vlincRNAs common to different cell lines, i.e. having a low K562-specificity index, had a higher degree of apoptosis (Figure 5A). The Spearman correlation between the cell specificity index (defined as the ratio of K562 levels to maximum levels in non-K562 tissues and the percentage of apoptotic cells) was -0.69, indicating that siRNAs against less K562-specific vlincRNAs had more effect in this study. Considering that K562-specificity of vlincRNAs is a totally independent parameter from the siRNA design and any potential off-target effects, the presence of this correlation strongly argues against a biological artifact in these assays. Rather, as one might expect, more constitutively expressed transcripts may have more basic function and thus a screen based on a basic phenotype, such as apoptosis would more readily reveal their phenotypes. More cell-type specific vlincRNAs, like those driven by LTRs, could have a more specialized function tuned to a specific cell-type, and thus result in more subtle or specialized phenotypes not assayed here.

Alternatively, the high level of expression of the K562-specific vlincRNAs could help explain why some of them did not show a significant phenotype. The Spearman correlation between the density of RNAseq signal in K562 and the percentage of apoptotic cells after siRNA treatment was -0.61. Depletion of the more highly expressed vlincRNAs might have less effect because sufficient levels could remain for function. Regardless, 3/10 K562-specific vlincRNAs had statistically-significant effect in this study and all 3 were driven by LTR promoters (Figure 5A; Additional File 3, Table S3 and Additional File 2, Figures S5-S9).

We measured the levels of depletion of vlincRNAs in 4 siRNA treatments that yielded the increased apoptosis phenotype, and in two that did not, using real-time RTPCR directly from sorted GFP-positive cells (Figure 5B, Materials and Methods, Additional File 4, Table S5). As expected, all 4 of the siRNAs that resulted in the increased apoptosis phenotype showed approximately 2-fold depletion of the corresponding RNAs. Of the two siRNAs that showed no phenotype, one resulted in depletion of the vlincRNA and one did not (Figure 5B). It is therefore possible that failure to observe phenotypes with some siRNAs could be due to inefficient depletion of the corresponding RNAs, or that a particular vlincRNA is not involved in the regulation of apoptosis.

To confirm that the observed phenotype was not due to non-specific events, we have selected two additional siRNAs per vlincRNA (Additional File 5, Table S4) for 6 vlincRNAs for which phenotype could be observed, 3 of which were LTR-driven, and repeated the experiments. All 12 siRNAs yielded the increased apoptotic phenotype (Figure 5C, Additional File 3, Table S3). The efficiencies of additional siRNAs varied, but the general trend remained the same: the correlation between fractions of apoptotic cells in the original siRNA experiments and the maximum of the two new siRNAs was 0.5.

### VlincRNAs vs lincRNAs

Overall, there is a low overlap between vlincRNAs and lincRNAs reported by Kelley and Rinn [23] at the base pair level: of the 279,818,144 base pairs covered by vlincRNAs, only 4,387,800 (1.56%) are covered by exons of lincRNAs. Median coverage of a vlincRNA that overlaps exons of a lincRNA is 1.4% of the vlincRNA length. Of the 9,241 lincRNAs, 1,137 (12.3%) overlap vlincRNAs. 1,232/2,147 (57.4%) of our vlincRNAs have no overlap with any lincRNA, and the 5' ends of 82.2% of strand-specific vlincRNAs were outside of 5 kb of a 5' end of a lincRNA. As discussed above, vlincRNA promoters contain different types of LTR elements and show greater variation between cell lines than those of lincRNAs. Also, vlincRNAs on average cover a much larger span of genome than lincRNAs (including the introns of the latter): median genomic length of 83,360 bp for a vlincRNA vs 10,686 bp for a lincRNA. The construction of vlincRNAs occurred based on either total cellular RNA or nuclear polyA-RNA, while lincRNAs were assembled primarily based on polyA+ RNA data. As such, the latter would be heavily enriched in spliced and polyadenylated non-coding RNAs. On the other hand, vlincRNAs would represent either polyA+ or polyA-RNAs.

However, even when vlincRNAs and lincRNAs overlap, there is still a question of which RNA functions in the cell, the lincRNA version, the vlincRNA version, or both. At least three lines of arguments suggest that the long ("vlincRNA-type") forms have function. First, the known and characterized macroRNAs function as long unspliced transcripts. Even though a spliced form for one such transcript, *Air*, exists, it makes up a minor form relative to the unspliced form [14,15]. Second, if unprocessed forms of lincRNAs are important, one would expect higher relative abundance of their intronic regions relative to exons compared to the known protein-coding genes. To assess this, we calculated the intronic and exonic densities of 781 lincRNAs overlapping K562 vlincRNAs and 43,958 UCSC transcripts expressed in that cell line. Using SMS RNAseq from total K562 RNA, we found that the median intron/exon ratio for lincRNAs was indeed significantly higher than for UCSC transcripts: 43.8% vs 12.6%. Finally, we have characterized a vlincRNA important in cellular senescence and have shown that its function depends on the unspliced isoform despite the fact a spliced form has been annotated (Lazorthes et al, manuscript in preparation).

## Discussion

In this study, we show that transcription from very long stretches of genomic space, often devoid of any known genes, exists as a common and yet un-recognized property of human cells. Extrapolating from the set of samples used here to identify vlincRNAs, this class of transcript could have much greater numbers and importance than previously anticipated. In fact, it's conceivable that by profiling a larger number of cell types, we will find that vlincRNAs emanate from most of the genome. Length stands out as a unique property of these sequences, with some of them reaching over 1MB (see Additional File 1, Table S1). At least in some cases, the transcripts are made as one whole RNA molecule (see Additional File 2, Figure S3; Lazorthes et. al., manuscript in preparation). As we show here, elements bearing chromatin marks of *bona fide *human promoters appear to regulate these vlincRNA regions. Notably, vlincRNAs encode functional RNA species: depletion of vlincRNA transcripts in this study has measurable phenotypic effects. In addition, all human samples profiled to date express vlincRNAs, including normal primary and embryonic stem cells and a normal human tissue, blood. Still, it is worth noting here that most of our conclusions were obtained utilizing a limited number of cell lines, and thus need to be further validated on an expanded set of primary tissues, both normal and cancerous.

VlincRNAs show statistically-significant association with SNPs associated with diseases in GWAS studies. In this respect, the recent work of van Dijk et. al. implicates a 205 kb vlincRNA to HELLP syndrome [30]. This HELLP vlincRNA completely overlaps one of our vlincRNAs, however our data suggests a longer and more complex locus (data not shown, see Additional File 1, Table S1). Remarkably, that study showed that point mutations linked to the disease could affect stability of the vlincRNA and result in downstream functional consequences. These findings should signal re-evaluation of the results of GWAS studies where disease-associated variants are often found outside of known genes.

All this begs the question of what such huge transcripts could be doing. While some of these vlincRNA regions could possibly produce spliced protein-coding transcripts, the long un-spliced versions of these RNAs would very unlikely code for proteins, especially considering that, for the most part, they localize to the nucleus (data not shown). Instead, these RNAs likely participate in the regulation of gene expression. Indeed, one such vlincRNA was shown to be critical for maintenance of the senescent state probably by altering expression of neighboring genes via epigenetic control of chromatin state (Lazorthes et. al., manuscript in preparation). These properties make vlincRNA regions similar to macroRNAs which were previously associated only with the regulation of imprinting [14,15]. However, until now, studies have recognized the presence of only a handful of such macroRNAs, limited to just six imprinted loci (*Igf2r, Igf2, Kcnq1, Pws/As, Gnas *and *Dlk1*) in the human and/or mouse genomes [14,15]. Our results suggest that gene regulation via vlincRNAs and macroRNAs could be a general theme in human cells. As recently suggested, these transcripts may function as a type of scaffold to facilitate regulatory or other processes happening in the nucleus, [31]. In this respect, the abundance of these transcripts relative to annotated coding mRNAs is noteworthy. Either the relative ratio of molecules of the two types of RNA, or mass of RNA represented by them can describe their abundance. If these very long molecules function as complex scaffolds, then the amount of RNA (mass) is also an important measure of their importance in a cell. We estimate that in K562 the average molar ratio of vlincRNA transcripts is 10-20 times lower than that of protein-coding transcripts, however, the average absolute mass of vlincRNAs is roughly 5 times higher than protein-coding transcripts.

Perhaps the most striking result comes from the association of tissue-specific vlincRNAs and LTR-based promoters. While cell-type specific promoters in general tend to be somewhat enriched in LTR sequences, the promoters of cell-type specific vlincRNAs demonstrate a much greater enrichment. The number of LTR-driven vlincRNAs correlates with certain cellular states. Specific activation of these very long non-coding transcripts controlled by LTR-based promoters connects stem cells with cancerous cells, both sharing a high potential for cell division. In this respect, it's reminiscent of very recent studies showing the prominent importance of cancer stem cells in disease [32-34]. It's tantalizing to speculate that these RNAs could participate in the complex machinery that supports the pluripotent-like properties of cancerous cells.

Activation of ERVs in some cancers has been observed previously. These studies have focused mostly on RNAs and proteins encoded by ERVs [35,36]. However, no connection has been made to the global importance of cellular non-coding RNAs, particularly vlincRNAs, driven by promoters embedded in ERVs or their LTRs in disease. Here we provide evidence that these cellular transcripts, rather than the LTRs themselves, constitute another component mediating the function of ERVs in pluripotency and malignancy. Exaptation of endogenous LTRs to serve as primary or alternative promoters of known genes is a well-documented phenomenon [22]. However, it seems that in most of the well characterized cases, the LTR promoter either contributes a minor effect in alternative regulation of the target gene or recapitulates the expression pattern conferred by the primary promoter [22]. The notable exception to this rule is specific redirection of expression to placenta [22]. Our results seem to suggest yet another paradigm, where the endogenous LTRs were exapted to serve as cell-type specific primary promoters of a class of non-coding RNAs, and this effect is most pronounced in pluripotent and malignant cells.

A general theme in the LTR transcriptional activation observed here is that only a very specific subset of LTRs function as promoters in any given cell line (Table 2), arguing against a genome-wide global activation of this type of sequence. For example, stem cells themselves could theoretically produce non-functional vlincRNAs due to their more open chromatin structure [37]. However, only a tiny fraction (< 0.1%) of all LTR sequences are active promoters specific to the H1-hEsc cell line, and only a fraction of those drive vlincRNA expression - a situation very different from non-specific, genome-wide activation of transcription. Still this activation is non-random as indicated by detection of vlincRNAs and their promoters in different biological replicas of the same cell line using different techniques. While we have shown that LTR-promoters of vlincRNAs do indeed have strong promoter activity in reporter-gene assays, their DNA sequence alone does not recapitulate the cell-type specificity found *in vivo*. Thus, it's likely that some other mechanisms such as epigenetic control define the specificity of LTRs. In fact, the epigenetic status of tissue or cell specific LTR promoters has been shown modulate their activation and repression [38-42]. Furthermore, tumor-specific alterations in DNA methylation of some repeat elements have been reported [43].

Finally, our results connect two genomic features typically considered "junk DNA": endogenous viral sequences, specifically LTRs, and non-coding, intergenic portions of the genome often with little annotation, the so-called "gene desert" regions. However, their specific activation in pluripotent and cancer cells combined with siRNA-based studies suggest that the conventional wisdom of ignoring these features should be revisited. Interestingly, enrichment of different LTR elements in vlincRNA promoters distinguishes the various cell lines. Even with the small number of cell lines analyzed here, some clear differences emerge. The reason behind these differences and their biological implications offers an interesting direction for future investigation.

## Conclusions

**Figure 6:**
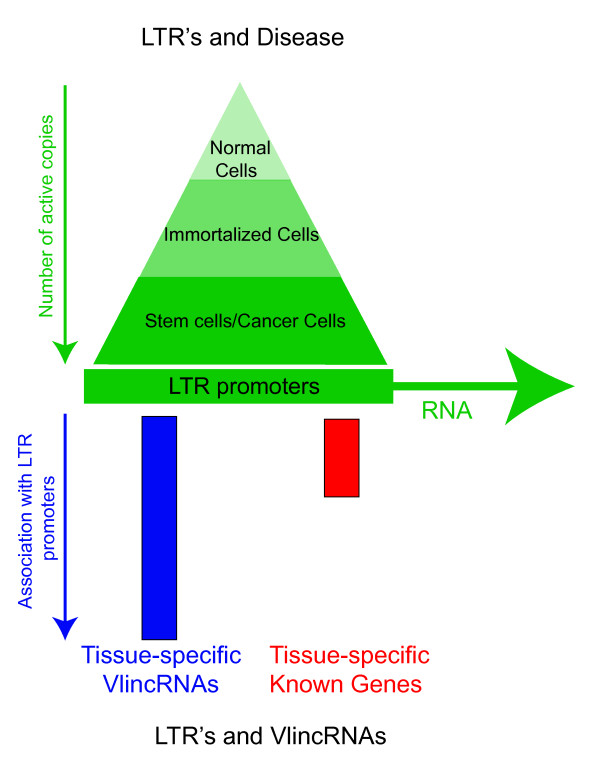
**Schematic diagram of two-dimensions of endogenous LTR promoter activity: gradient of activation in malignant/pluripotent state and preferential association with tissue-specific vlincRNAs**.

We show two potentially independent dimensions of functional, endogenous LTRs in the human genome: selective activation of promoter capabilities of a specific subset of LTRs in stem and cancerous cells, and association of LTR promoters with a specific functional class of very long non-coding RNAs, vlincRNAs (Figure 6). These intriguing connections shine a different light on ignored portions of the genome and highlight the question of why specific subsets of LTRs and vlincRNAs activate in very specific stages of development.

Given the high abundance of vlincRNAs shown in this paper, we could speculate that much of the dark matter RNA may take the form of these huge transcripts. While the scientific community continues to look at the genome through the prism of short ESTs or lincRNAs scattered in intergenic space, our data suggests that many of these transcripts actually belong together in very long transcripts regulated by relatively few promoters. This observation could be an organizing theme in the biology of non-coding RNA regulation: very long RNAs that traverse our genome may regulate gene expression in the nucleus, spiking in early development, and capable of reactivation in the malignant cellular state.

## Materials and methods

Datasets used

I. Publicly-available

1. **The 580 K562 - Ewing Sarcoma VlincRNAs**: the coordinates of the vlincRNAs are listed in the Supplementary Table S3 [12].

2. **Promoters from 9 human cell lines **annotated by the ENCODE consortium, Broad Institute, MIT and MGH [5,16,17,44]. The basis of this analysis was profile of 9 different chromatin marks and input controls in 9 human cell lines. A Hidden Markov Model was then used to define 15 different chromatin states based on the different marks, including 3 states that corresponded to Active, Weak and Poised promoters [17].

3. **ENCODE Yale/UC-Davis/Harvard Transcription Factor Binding Sites **[5,21,45].

4. **ENCODE/CSHL long nuclear polyA- and total polyA-RNAseq dataset **[5,6,24,46].

5. **LTR repeats **were extracted from the RepeatMasker track tables chr[N]_rmsk.txt, where N = 1, ..., 22, X, Y, M [47,48].

6. **UCSC Genes annotations **were from the knownGene.txt file: HG19 [47] (Last modified 05-Feb-2012) and HG18 [48] (Last modified 10-May-2009).

7. **Disease-associated SNPs from GWAS studies **were downloaded from the corresponding track on the UCSC Genome browser [49].

8. **Alignability track **(36mers, Guigo - CGR, Barcelona) was downloaded from the UCSC browser [50].

II. Un-published

9. **Human blood SMS RNAseq dataset**. Prior to routine angiographic testing for coronary artery disease, adult volunteers were consented and peripheral blood was drawn under IRB-approved protocols. All human studies were conducted in accordance with the principles of the Helsinki Declaration. All patients provided informed consent under protocols approved by The George Washington University Institutional Review Board (#111051). Blood was drawn into Tempus blood RNA stabilization tubes and stored at -80°C until blood RNA was isolated by pelleting cells, disruption, and column capture of the nucleic acids. Aggressive DNAse treatment was conducted by treating 10 ug of total RNA with 10 ul TURBO DNase Buffer and 2 ul Turbo DNase (Invitrogen AM2238) in a total volume of 100 µl at 37°C for 30 minutes followed by DNAse removal by affinity capture. The resulting RNA was cleaned using the Qiagen RNAeasy MinElute Cleanup Kit (Qiagen). Up to 5 ug of the resulting RNA was then depleted of ribosomal RNA using the RiboZero rRNA Removal Kit (Epicentre) following the manufacturer's protocol, typically yielding 300-400 ng of rRNA-depleted RNA. cDNA preparation, tailing and sequencing using Helicos single-molecule sequencing technology was performed essentially as described [12,51]. In total, ~120 million informative reads were produced. The latter were defined as reads uniquely aligning to the genome with a minimal normalized score [12] of 4.3 and filtered for rRNA and chrM reads.

10. **Mouse inflammation RNAseq dataset **was produced by combining ~1 billion informative reads generated from rRNA-depleted total RNA isolated from control mouse lung tissue and also treated with lipopolysaccharide in different regimes using Helicos single molecule sequencing technology. The experimental details and other aspects of this dataset are described in St. Laurent et al [52]. The informative reads were defined as reads uniquely aligning to the genome with a minimal normalized score [12] of 4.3 and filtered for rRNA and chrM reads.

The filtered relevant reads from the above datasets were posted in SRA [SRA:SRP021192].

### Construction of vlincRNA regions from different RNAseq datasets

The ENCODE/CSHL long RNAseq data from nuclear polyA-RNA subcompartment was downloaded in the form of "contigs" that represent blocks of overlapping mapped reads from the pooled biological replicates. We removed any contigs that overlapped exons or introns of UCSC Genes. Since the data is strand-specific, all downstream analysis was done in a strand-specific fashion - contigs that overlapped genes on the other strand were not removed. Contigs separated by 1000 bp or less were merged and only those merged contigs that were at least 50 kb in length were allowed. Those separated by 5 kb or less were merged together. The resulting vlincRNAs were strand-specific.

For construction of vlincRNAs from blood and mouse, we have first created the read density for each genomic base in non-strand specific fashion, then removed positions corresponding to annotated genes. We then applied to the following consecutive thresholds: density threshold corresponding to the 80^th ^%-ile of expression, followed by merging genomic bases separated by no more than 500 bp (blood) or 1000 bp (mouse), followed by selection of regions of 50 kb or longer. The resulting vlincRNAs were not strand-specific. For comparison with the human vlincRNAs, the coordinates of the mouse vlincRNAs were converted to human coordinates using the LiftOver tool from the UCSC Genome Browser [53].

Overlap between different datasets

I. Overlap and p-value calculation between the strand-specific vlincRNAs and promoters.

1) When appropriate, Active, Weak and Poised Promoters from each of the 6 cell lines (K562, GM12878, HepG2, HUVEC, NHEK and H1-hEsc) were overlapped with LTR repeats. In such cases, only promoters that overlap LTR repeats were selected.

2) 10 kb intervals one for each vlincRNA from each of the 6 cell lines were constructed (+/-5 kb from left border for the top strand vlincRNAs and +/-5 kb from right border for the bottom strand vlincRNAs).

3) The number  n of 10 kb vlincRNA intervals that overlap promoters was calculated for each cell line and each strand.

4) Probability $p_{i}$ that 10 kb interval of  i-th vlincRNA overlap a promoter was calculated by formula

$$p_{i}=\left|Occupied\text{\_}space_{i}\right|/\left(|Space_{i}|-V\right),$$

for each i=1,…,N, where

 N - number of vlincRNAs in a given dataset and given strand, |X| is the operator for taking the total length of the intervals *X*

$Space_{i}$ = Genomic space **minus **Anti_space intervals whose left boundaries for top stranded vlincRNAs (right boundaries for bottom stranded vlincRNAs) were extended by length of the given vlincRNA,

Anti_space = genomic intervals occupied by UCSC Known Genes or Encode blacklisted regions* **minus **parts that overlap tested vlincRNAs. Gene on the opposite strand was considered intergenic.

 V = total length of tested vlincRNAs ( V subtracted from $\left|Space_{i}\right|$ to account for multiple number of vlincRNAs tested and make upper bound estimation of p-value).

$Occupied\text{\_}space_{i}=Space_{i}$ covered by the tested promoters extended by 5 kb on each side from the corresponding cell line.

*UCSC accessions wgEncodeEH001432 and wgEncodeEH000322.

5) The expected number  m of intervals overlapping promoters for each cell line and each strand was calculated as:

$$m=\sum_{i=1}^{N}p_{i}.$$

6) P-value as a probability P(ξ≥n) was calculated under assumption that ξ (random variable stand for number of vlincRNAs that 10 kb interval overlap a promoter) distributed as binomial $B\left(N,\frac{m}{N}\right)$.

II. Overlap and p-value calculation between the non-strand specific vlincRNAs and promoters.

1) For each vlincRNA in a test with size ***N***, two 10 kb intervals were constructed: +/-5 kb from the left boundary and +/-5 kb from the right boundary.

2) The number of vlincRNAs ***n***with at least one of the two 10 kb intervals overlapping a promoter from the tested class was determined.

3) Probability $p_{i}$ that at least one of two 10 kb intervals of  i-th vlincRNA overlap a promoter was calculated by formula

$$p_{i}=1-\left(1-p_{i}^{+}\right)\left(1-p_{i}^{-}\right),$$

$$p_{i}^{+}=\left|Occupied\text{\_}space_{i}^{+}\right|/\left(|Space_{i}^{+}|-V\right),$$

$$p_{i}^{-}=\left|Occupied\text{\_}space_{i}^{-}\right|/\left(|Space_{i}^{-}|-V\right),$$

for each i=1,…,N, where

$Space_{i}^{+}$ = Genomic space **minus **Anti_space intervals whose left boundaries were extended by length of the given vlincRNA,

$Space_{i}^{-}$ = Genomic space **minus **Anti_space intervals whose right boundaries were extended by length of the given vlincRNA,

Anti_space = genomic intervals occupied by UCSC Known Genes on either strand or Encode blacklisted regions **minus **parts that overlap tested vlincRNAs,

 V = total length of tested vlincRNAs,

$Occupied\text{\_}space_{i}^{+/-}=Space_{i}^{+/-}$ covered by the tested promoters extended by 5 kb on each side from the corresponding cell line.

4) Expected number of vlincRNAs ***m***with at least one of the two 10 kb intervals overlapping a promoter from the tested class was calculated as:

$$m=\sum_{i=1}^{N}p_{i}.$$

5) P-value as a probability P(ξ≥n) was calculated under assumption that ξ distributed as binomial $B\left(N,\frac{m}{N}\right)$.

III. Calculation of p-value for how many vlincRNAs from set 1 overlap vlincRNAs from set 2.

This procedure is applicable for any genomic interval data in set 2 including SNP.

1) Probability $p_{i}$ that  i-th vlincRNA from set 1 overlap any vlincRNA from set 2 was calculated by formula

$$p_{i}=\left|Occupied\text{\_}space_{i}\right|/\left(|Space_{i}|-V\right),$$

for each i=1,…,N, where

 N - number of vlincRNAs in set 1,

$Space_{i}$ = Genomic space **minus **Anti_space intervals whose both boundaries were extended by half of length $L_{i}/2$ of the given vlincRNA,

Anti_space = genomic intervals occupied by UCSC Known Genes or Encode blacklisted regions **minus **parts that overlap vlincRNAs from set 1.

 V = total length of vlincRNAs from set 1.

$Occupied\text{\_}space_{i}=Space_{i}$ covered by the vlincRNAs from set 2 whose both boundaries were extended by $L_{i}/2$.

2) Expected number ***m***of vlincRNAs from set 1 overlapping any vlincRNA from set 2 was calculated as:

$$m=\sum_{i=1}^{N}p_{i}.$$

3) P-value as a probability P(ξ≥n), where  n is actual number of vlincRNAs from set 1 overlapping vlincRNAs from set 2, was calculated under assumption that ξ distributed as binomial $B\left(N,\frac{m}{N}\right)$.

IV. Non-strand-specific overlap and p-value calculation between LTRs and vlincRNA promoters.

1) VlincRNA promoters were defined as a join of Active, Weak and Poised promoters in each cell line separately (K562, GM12878, HepG2, HUVEC, NHEK and H1-hEsc) that overlap 10 kb intervals that center is a 5' edge of a vlincRNA of the same cell line.

2) Several LTRs of the same type (LTR7, HERVH-int,...) were collapsed into one cluster if they were located in the same promoter or in promoters of the same vlincRNA. If an LTR overlaps two promoters, it is split into two LTRs before assigning it to a cluster. LTR clusters are assumed to be distributed in genome independently relative to each other.

3) The number  n of LTR clusters that overlap vlincRNA promoters was calculated for each cell line and each LTR type in non-strand-specific manner.

4) Probability  p that an LTR cluster overlaps a vlincRNAs promoter was calculated by formula

$$p=\left|Vlincs\text{\_}promoters\right|/\left|Space\text{\_}promoters\right|,$$

where

|⋅| - operator for taking of the total length of intervals,

Space_promoters - promoters that overlap Space,

Space = Genomic space **minus **Anti_space intervals whose both boundaries were shrunk by 5 kb,

Anti_space = genomic intervals occupied by UCSC Known Genes **plus **genomic intervals shorter than 50 kb between UCSC Known Genes **minus **parts that overlap vlincRNAs from all 6 cell lines.

5) The number  N of LTR clusters overlapping Space_promoters was calculated for each LTR type.

6) P-value as a probability P(ξ≥n) was calculated under assumption that ξ (random variable stand for number of LTR clusters overlap vlincRNA promoters) distributed as binomial B(N,p).

V. Strand-specific overlap and p-value calculation between LTRs and vlincRNA promoters.

1) VlincRNA promoters were defined as in IV (1).

2) LTRs on the top strand were collapsed into clusters as in IV (2) and LTRs on the bottom strand were collapsed into clusters in the same way.

3) The number $n_{+}\left(n_{-}\right)$ of top (bottom) strand LTR clusters that overlap top (bottom) strand vlincRNA promoters was calculated for each cell line and each LTR type.

4) Probability $p_{+}\left(p_{-}\right)$ that a top (bottom) strand LTR cluster overlaps a top (bottom) strand vlincRNAs promoter was calculated by formula

$$p_{+}=\left|Vlincs\text{\_}promoters_{+}\right|/\left|Space\text{\_}promoters_{+}\right|,$$

$$p_{-}=\left|Vlincs\text{\_}promoters_{-}\right|/\left|Space\text{\_}promoters_{-}\right|$$

where

 - operator for taking of the total length of intervals,

$Vlincs\text{\_}promoters_{+\left(-\right)}$ - promoters of top (bottom) strand vlincRNAs,

$Space\text{\_}promoters_{+\left(-\right)}$ - promoters that overlap $Space_{+\left(-\right)}$,

$Space\text{\_}promoters_{+\left(-\right)}$ = Genomic space **minus **$Anti\text{\_}space_{+\left(-\right)}$ intervals whose both boundaries were shrunk by 5 kb,

$Anti\text{\_}space_{+\left(-\right)}$ = genomic intervals occupied by top (bottom) strand UCSC Known Genes **plus **genomic intervals shorter than 50 kb between top (bottom) strand UCSC Known Genes **minus **parts that overlap top (bottom) strand vlincRNAs from all 6 cell lines.

5) The number $N_{+}\left(N_{-}\right)$ of top (bottom) strand LTR clusters overlapping $Space\text{\_}promoters_{+\left(-\right)}$ was calculated for each LTR type.

6) Upper bound of p-value as a probability $P\left(\xi \geq n_{+}+n_{-}\right)$ was calculated under assumption that ξ (random variable stand for number of LTR clusters overlap vlincRNA promoters) distributed as binomial $B\left(N_{+}+N_{-},\text{max}\left\{p_{+},p_{-}\right\}\right)$.

#### Long-range PCR Assays

RT-PCR

First strand synthesis was performed using Superscript III (Invitrogen, 18080-051) following the manufacturer's protocols with 200 ng Total DNA-free K-562 RNA (Agilent, 540103) as a template. See the Additional File 6, Table S2 for the gene-specific RT primers used. Each sample had RNA removed with the addition of 1 µl RNAse H, incubated at 37 °C for 30 minutes. Subsequent PCR was performed using Long Amp Taq PCR Kit (NEB, E5200S) following the manufacturer's protocol on 2.5 µl cDNA in a 25 µl final volume (94 °C 30s, 34x(94 °C 30 s, 55 °C 30s, 65 °C 5 m), 65 °C 10 m). See the Additional File 6, Table S2 for the PCR primers used and the coordinates of the PCR regions. Products were run on 1% agarose gel.

Genomic PCR

500 ng of genomic DNA from K562 cell line (Promega, DD201A) was used for PCR using the conditions above.

#### Luciferase Reporter Gene Assays

Genomic DNA extracted from cultured cells (Hep3B) using a kit from Promega was used as template to amplify the vlincRNA promoter regions detailed below.

Vlinc_185 range_hg18=chr17:65636584-65637598 (**V185**).

Vlinc_203 range_hg18=chr18:73834656-73835677 (**V203**)

PGL3-basic-V185(1015bp) was generated by PCR and subcloned into the PGL3-basic vector at *Sac*I-*HindII*I sites, the forward primer was CC**GAGCTC**TTGAATTCCAGGGGTACCAG and the reverse primer was CCC**AAGCTT**AGACGGGCTGAGGTCTACAA;

PGL3-basic-V203(1022bp) was generated by PCR and subcloned into the PGL3-basic vector at *Kpn*I-*HindII*I sites, the forward primer was GG**GGTACC**CTGCCAAGGTGAAAGATGCT and the reverse primer was CCC**AAGCTT**GCAGGCAAAAAGAGCCTATG;

Transient transfection was performed with Lipofectamine 2000 reagents (Invitrogen) by following the low serum protocol recommended by the manufacturer. Hep3b cells were trypsinized 24 hours after transfection, pooled, divided equally, and cultured in 12-well plates for luciferase assays. The pRL-CMV (0.1 μg) was cotransfected to normalize the transfection efficiency. After 24 hours transfection, suspension K562 cells were pooled, divided equally, and cultured in 12-well plates for reporter assays. Luciferase and dual luciferase assays were performed in 6 replicas for Hep3b and 3 replicas for K562 with kits from Promega. Luminescence was measured in a TD20/20 luminometer (Turner Biosystems), and relative light units (RLU) were standardized to *Renilla *luciferase activity expressed from cotransfected pRL-CMV.

### Quantitation of vlincRNA expression in 16 ENCODE cell lines

The ENCODE/CSHL long RNAseq data from total polyA-RNA was downloaded in the form of "contigs" that represent blocks of overlapping mapped reads from the pooled biological replicates. The 16 cell lines were: A549, AG04450, BJ, CD20+, GM12878, H1-hESC, HeLaS3, HepG2, HMEC, HSMM, HUVEC, K562, MCF7, Monocytes-CD14+, NHEK, NHLF and SK-N-SH (treated with retinoic acid). More information about the cell lines can be found on the UCSC browser at the link for this dataset above. For each vlincRNA, the counts based on total number of bases covered by reads from the contig data (column 9 of the BED files) were obtained and normalized to 20G bases and and 1MBp of vlincRNA length. Individual vlincRNAs built from the 6 ENCODE cell lines (K562, HepG2, GM12878, H1-hESC, NHEK and HUVEC) that could be assigned to a promoter in All category (see Additional File 1, Table S1) were used in this analysis. These vlincRNAs were further stratified into 468 and 739 vlincRNAs that could be assigned to a promoter that overlapped or not an LTR sequence. The counting was done in a strand-specific fashion and contigs with very high counts (> 1M) were removed as they could likely represent artifacts or alignments to highly expressed sequences such as snRNA or rRNA repeats. Maximal values for the cancerous and primary cell lines were calculated (the immortalized and ESC cell lines were represented only by a single cell line each) for each vlincRNA. The ratio of maximal values in cancerous cell lines vs that in primary was then calculated as well as the ratios of immortalized to max primary and ESC to max primary. Zero values were replaced by the minimal non-zero expression value in the dataset. The averages of these ratios are shown in the Table 4.

#### VlincRNA inhibition experiments using RNAi

siRNAs against 20 vlincRNAs of the original 580 vlincRNAs published in Kapranov et al [12], 1 siRNA per vlincRNA, were designed using the Whitehead siRNA design tool [54] and synthesized by Sigma-Aldrich with dTdT overhangs (sense and antisense). The design was done to limit possible off-target effects by selecting siRNAs with smallest possible number 7mers shared with conserved regions in 3' UTRs of known genes [54] (see Additional File 5, Table S4). In addition one negative control siRNA, MISSION siRNA universal negative control #1, (Sigma-Aldrich) and two positive controls were used: one positive control was the published siRNA against BCR-ABL fusion mRNA[55] and another was proprietary "AllStars Hs Cell Death Control siRNA" mix of siRNAs against house-keeping human genes purchased from Qiagen. In addition, for 6 vlincRNAs that showed phenotypes in the first of the siRNA experiments, we designed two additional siRNAs per vlincRNA (see Additional File 5, Table S4).

K562 cells were cultured in RPMI-1640 media with 10% fetal bovine serum, 100 µg of penicillin/ml, 100 U of streptomycin/ml. The cells were maintained in a humidified 37°C incubator with 5% CO_2_. For transfection, 1x10^5 ^cells were seeded in a well of 6-well plate with antibiotics-free media overnight. Then 50 nM siRNA (final concentration) and 1 µg pAAV-CB-EGFP plasmid were complexed with 6 µl of Lipofectamine 2000 (Invitrogen) in 500 µl of Opti-MEM media (Invitrogen) according to the manufacturer's instruction. The cells were collected 48 hours after transfection and pelleted by centrifugation at 500g for 5 minutes. The cells were washed once using 800 µl of labeling buffer (10 mM HEPES buffer with pH 7.4, 2.5 mM CaCl_2 _and 140 mM NaCl). The cells were resuspended in 300 µl of Annexin V-APC (eBiosciences) labeling buffer and incubated on ice for 15 min. After washing twice using the labeling buffer, the cells were stained in 300 µl of 7-AAD (Invitrogen) labeling buffer and incubated on ice for 2 minutes. The cells were washed once before suspended in the labeling buffer for flow cytometry analysis. For apoptosis analysis, 2,000 EGFP-expressing cells were counted by FACSCalibur flow cytometer Calibur (BD Biosciences) and the apoptotic cells were detected as being positive for both EGFP and annexin V. The statistical analysis were performed by one-tailed Student's t-test compared with the negative control siRNA. Statistically significance was defined as *P *< 0.05.

### Real-time RTPCR on siRNA-treated cells

1x10^5 ^of K562 cells were co-transfected with siRNA and pAAV-CB-EGFP plasmid as mentioned above. The transfections were duplicated for each siRNA. The cells were collected at 48 hours after transfection and washed twice with PBS. For each transfection, 2x10^4 ^cells expressing EGFP were sorted into a 1.5ml-Eppendorf tube containing 10ul of PBS via Influx™ cell sorter (BD Biosciences) and the final volume was 50 µl. 5 µl of the sorted EGFP cells were used for RNA extraction and reverse transcription using TaqMan® gene expression cells-to-CT TM kit according to the instruction. Real-time PCR reactions were duplicated for each RNA sample. The real-time reaction was performed in 20-µl system which contained 10 µl of 2x Fast SYBR® Green Master Mix (Invitrogen), 0.4 µl of each primer (100 µM), 4.2 µl of water and 5 µl of cDNA template. The real time reactions were run in Mastercycler® Realplex2 (Eppendorf) with the profile 95°C 20 sec, and 45 cycles of 95°C 3 sec and 60°C 30 sec. GAPDH was used as the normalization control. All primers are given in Additional File 4, Table S5.

## Abbreviations

CAGE: Cap Analysis of Gene Expression; CML: chronic myelogenous leukemia; ENCODE: Encyclopedia of DNA elements; ERV: endogenous retrovirus; LTR: long terminal repeat; SMS: single-molecule sequencing; vlincRNA: very long intergenic non-coding RNA; lincRNA: long intergenic non-coding RNA.

## Competing interests

The authors declare that they have no competing interests.

## Authors' contributions

D.S. and P.K. performed the statistical and bioinformatics analyses; B.D., X.F. and W.X. performed the siRNA/flow cytometry analyses; M.T. performed sample preparation and SMS sequencing; N.S. performed the reporter gene analyses; S.L. and E.N. extensively shared and discussed data; T.J.T. provided theoretical insights into ncRNAs and cancer; T.A.M. performed the human blood analysis and provided theoretical insights; P.K., G.S.L and W.X. provided the theoretical framework and guidance for the study and wrote the manuscript. All authors read and approved the final manuscript.

## Supplementary Material

Additional File 1 Supplementary Table S1 Click here for file

Additional File 2 Supplementary Figures S1-S9 Click here for file

Additional File 3 Supplementary Table S3 Click here for file

Additional File 4 Supplementary Table S5 Click here for file

Additional File 5 Supplementary Table S4 Click here for file

Additional File 6 Supplementary Table S2 Click here for file
