# Supplementary material for: VlincRNAs controlled by retroviral elements are a hallmark of pluripotency and cancer
Source: Genome Biol. 2013 Jul 22;14(7):R73. doi: 10.1186/gb-2013-14-7-r73 (PMC4053963; doi:10.1186/gb-2013-14-7-r73)
Supplement: Additional File 2 — Supplementary Figures S1-S9 [file gb-2013-14-7-r73-S2.PDF]

Supplementary Figure Legends

Figure S1. An example of a vlincRNA present in cancerous and normal embryonic stem cell lines. Panels B & C show zoom-in view on the corresponding promoter regions marked by arrows in A. The vlincRNA in the H1-hEsc cell line appears to be driven by a bi-directional promoter upstream of another non-coding RNA SNHG5 which is a host for two snoRNAs (A & B). The H1-hEsc vlincRNA also overlaps two vlincRNAs (vlinc_426 and vlinc_427) found by us previously in cancerous samples [1] and spanning ~74% of the region covered by the H1-hEsc vlincRNA (A). Expression of the H1-hEsc vlincRNA also occurred in the HUVEC normal primary cell line, as well as a cancerous cell line HepG2, but not in K562, NHEK or HeLa (data not shown). This region may harbor different transcripts: the K3K4Me3 histone mark typically found at active promoters can be seen inside the H1-hEsc vlincRNA domain and at the boundary of Vlinc_427, suggesting that an alternative initiation site within this region may exist and Vlinc_427 could be a stand-alone transcript unit (A). Furthermore, lower level transcription in H1-hEsc extended beyond the boundary of the vlincRNA (A) suggests that the size of that transcribed region could be much larger than 260 kb. RNAseq data shows density of informative reads normalized by 10M informative reads. The Y-axis of the alignability track (Materials and Methods) is on the scale of 0 to 1. Coordinates: hg19.

Figure S2. Alignability scores for all 2147 vlincRNAs and 1928 vlincRNAs after removal of 219 vlincRNAs with poor upstream alignability. See text and Materials and Methods for details.

Figure S3. Long-range RT-PCR analysis of contiguous transcripts in vlincRNA regions. Genomic view (A) and zoom-in on the 5' end (B) of a standalone LTR-driven K562 vlinc_228 (hg19 coordinates of chr2:76095748-76294624; Supplementary Table S1) is shown. The tile of overlapping long-range RT-PCR regions, vlinc228_-1 to vlinc228_+11 that span >50kb is shown (Materials and Methods; Supplementary Table S2). The first reaction (vlinc228_-1) spans region upstream of the ENCODE K562 nuclear polyA- RNAseq signal and as expected yielded no RT-PCR product while all other reactions were positive (C). The same primer pair yielded a positive product on K562 genomic DNA. The RT-PCR reactions were done with ("+" lanes) or without ("-" lanes) reverse transcriptase (C). RNAseq data shows density of aligned reads normalized. The Y-axis of the alignability track (Materials and Methods) is on the scale of 0 to 1. Coordinates: hg19.

Figure S4. Diagram of association between K562-specific vlincRNAs and UCSC transcripts with Active promoters and LTRs in their promoter regions. K562-specific vlincRNAs (blue box) and UCSC transcripts were selected on the basis of being >10 fold higher than in other tissues examined. 60 K562-specific vlincRNAs were further manually clustered based on EST evidence into 53 (blue box) and 496 K562-specific UCSC transcripts were collapsed to select 343 with unique 5' ends (green box). K562 Active promoters (AP) in cells and K562-

specific AP selected on the basis on non-overlap with any promoter in any of the 8 other ENCODE cell lines are shown as red boxes. The sets of promoters that overlap an LTR and those promoters where the overlap with an LTR is in the core of the promoter based on ENCODE TFBS data are shown as yellow boxes. The numbers of vlincRNAs (blue text), UCSC transcripts (green text) and Active Promoters (red text) are indicated for each set and overlapping subsets. For example, 34 K562-specific vlincRNAs can each be associated with a K562-specific AP that overlaps an LTR and reciprocally there are 39 such promoters. Also, see text for details.

Figure S5. Examples of flow cytometry profiles obtained in RNAi vlincRNA inhibition experiments: EGFP-only.

$1x10^5$ K562 cells were transfected with 50 nM siRNA (final concentration) and 1 µg pAAV-CB-GFP plasmid and collected for apoptosis analysis at 48 hr after transfection. Transient transfection for each siRNA was performed in triplicate and one was shown as representative. A. Based on forward scatter and side scatter, a morphologically normal cell population was gated for down-stream analysis. B. Percentage of apoptotic cells (Annexin V^+/7-AAD^-, lower) and late apoptotic cells (Annexin V^+/7-AAD^+, upper). C. Percentage of living GFP-expressing cells (GFP^+/7-AAD^-). D. Percentage of apoptotic cells within GFP-expressing cells (GFP^+/Annexin V^+/7-AAD^-), used as the index for the apoptotic inducing effect of siRNA in this study. A total of 2,000 GFP-expressing cells were counted.

Figure S6. Examples of flow cytometry profiles obtained in RNAi vlincRNA inhibition experiments: MISSION negative control #1.

See legend for Figure S5 for details.

Figure S7. Examples of flow cytometry profiles obtained in RNAi vlincRNA inhibition experiments: Positive control AllStars Hs cell Death Control siRNA.

See legend for Figure S5 for details.

Figure S8. Examples of flow cytometry profiles obtained in RNAi vlincRNA inhibition experiments: siRNA against vlinc_21 – example of no effect and vlinc_243 – significant induction of apoptosis.

See legend for Figure S5 for details.

Figure S9. Examples of flow cytometry profiles obtained in RNAi vlincRNA inhibition experiments: siRNA against vlinc_243 – significant induction of apoptosis.

See legend for Figure S5 for details.

1. Kapranov P, St Laurent G, Raz T, Ozsolak F, Reynolds CP, Sorensen PH, Reaman G, Milos P, Arceci RJ, Thompson JF, Triche TJ: **The majority of total nuclear-encoded non-ribosomal RNA in a human cell is 'dark matter' un-annotated RNA.** *BMC Biol* 2010, **8:**149.

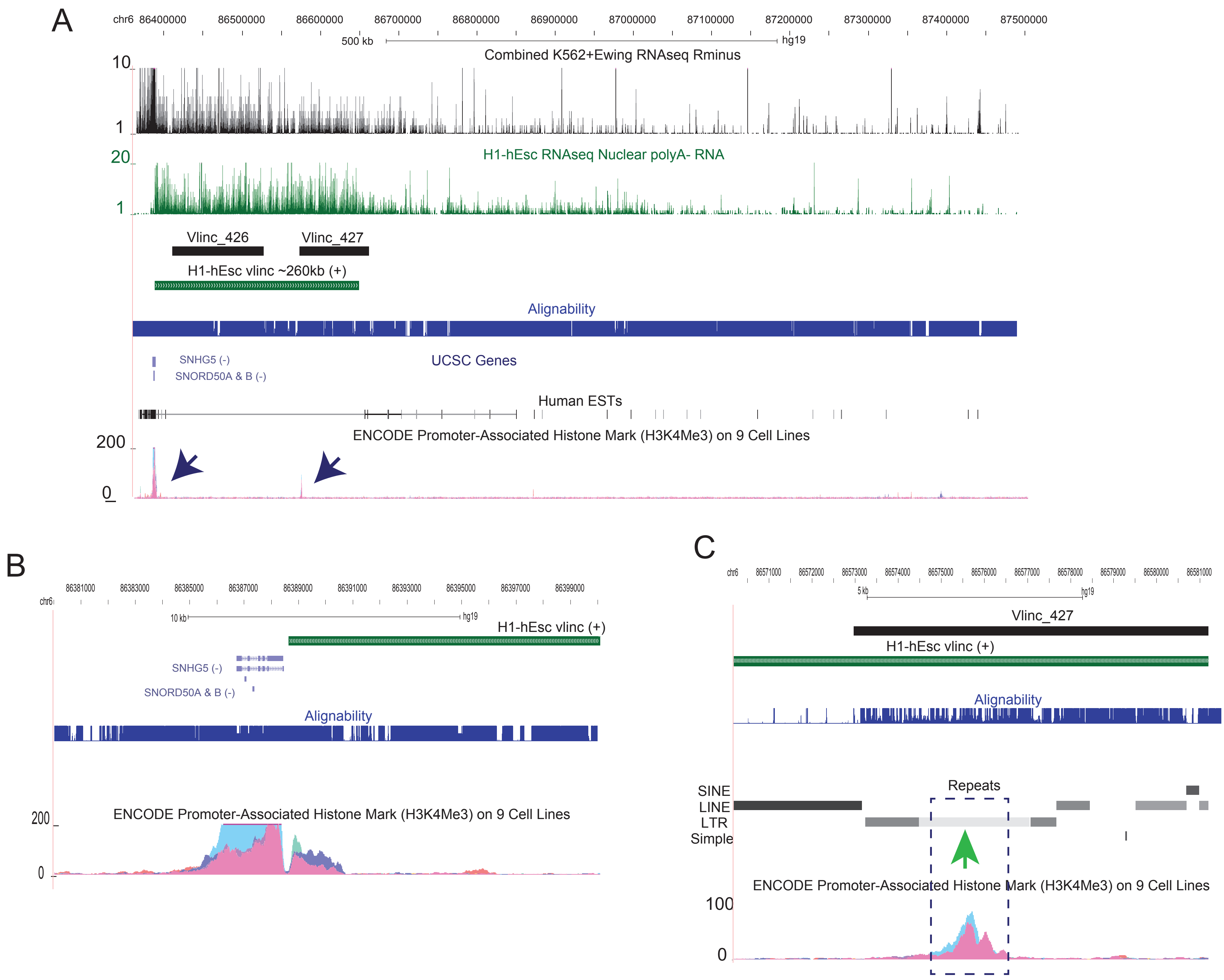
A
chr6 86400000 86500000 86600000 86700000 86800000 86900000 87000000 87100000 87200000 87300000 87400000 87500000
500 kb
hg19
Combined K562+Ewing RNAseq Rminus
10
1
H1-hEsc RNAseq Nuclear polyA- RNA
20
1
Vlinc_426
Vlinc_427
H1-hEsc vlinc ~260kb (+)
Alignability
SNHG5 (-)
SNORD50A & B (-)
UCSC Genes
Human ESTs
ENCODE Promoter-Associated Histone Mark (H3K4Me3) on 9 Cell Lines
200
0
B
86381000 86383000 86385000 86387000 86389000 86391000 86393000 86395000 86397000 86399000
chr6
10 kb
hg19
H1-hEsc vlinc (+)
SNHG5 (-)
SNORD50A & B (-)
Alignability
ENCODE Promoter-Associated Histone Mark (H3K4Me3) on 9 Cell Lines
200
0
C
chr6 86571000 86572000 86573000 86574000 86575000 86576000 86577000 86578000 86579000 86580000 86581000
5 kb
hg19
Vlinc_427
H1-hEsc vlinc (+)
Alignability
Repeats
SINE
LINE
LTR
Simple
ENCODE Promoter-Associated Histone Mark (H3K4Me3) on 9 Cell Lines
100
0

Figure S1

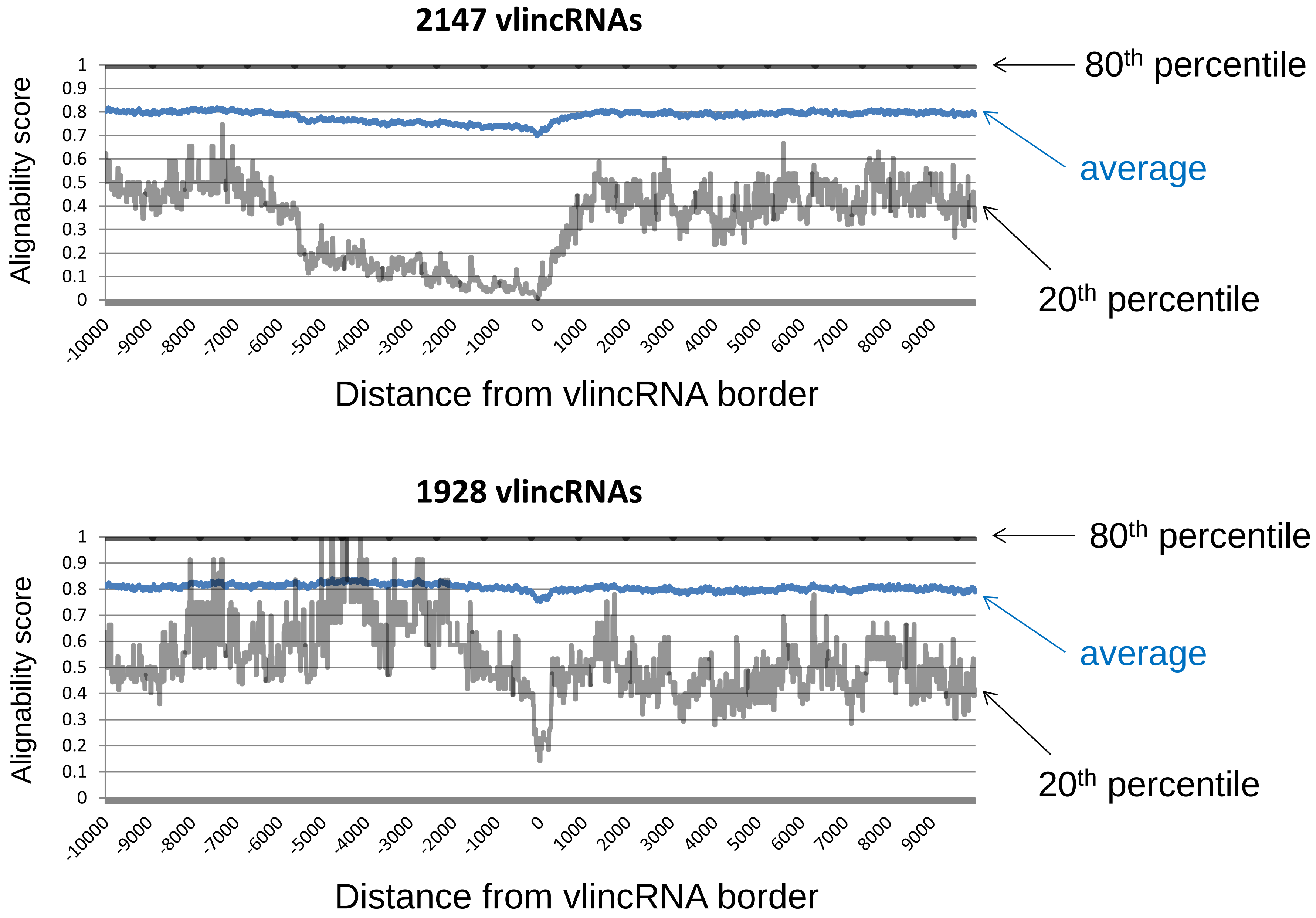
2147 vlincRNAs
Alignability score
1
0.9
0.8
0.7
0.6
0.5
0.4
0.3
0.2
0.1
0
-10000
-9000
-8000
-7000
-6000
-5000
-4000
-3000
-2000
-1000
0
1000
2000
3000
4000
5000
6000
7000
8000
9000
Distance from vlincRNA border
80th percentile
average
20th percentile
1928 vlincRNAs
Alignability score
1
0.9
0.8
0.7
0.6
0.5
0.4
0.3
0.2
0.1
0
-10000
-9000
-8000
-7000
-6000
-5000
-4000
-3000
-2000
-1000
0
1000
2000
3000
4000
5000
6000
7000
8000
9000
Distance from vlincRNA border
80th percentile
average
20th percentile

Figure S2

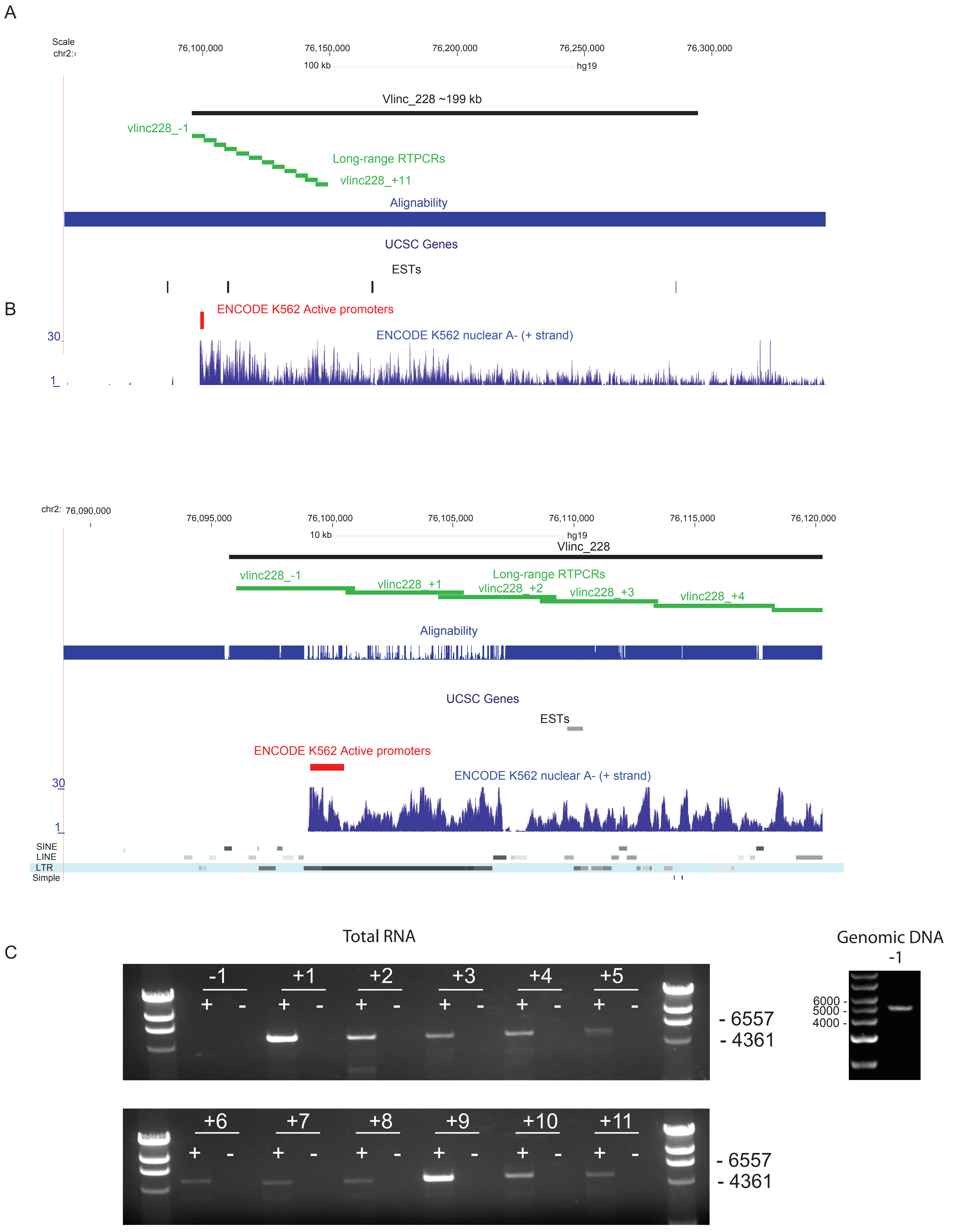
A
Scale
chr2:
76,100,000
76,150,000
76,200,000
76,250,000
76,300,000
100 kb
hg19
Vlinc_228 ~199 kb
vlinc228_-1
Long-range RTPCRs
vlinc228_+11
Alignability
UCSC Genes
ESTs
B
ENCODE K562 Active promoters
30
ENCODE K562 nuclear A- (+ strand)
1
chr2: 76,090,000
76,095,000
76,100,000
76,105,000
76,110,000
76,115,000
76,120,000
10 kb
hg19
Vlinc_228
vlinc228_-1
vlinc228_+1
Long-range RTPCRs
vlinc228_+2
vlinc228_+3
vlinc228_+4
Alignability
UCSC Genes
ESTs
ENCODE K562 Active promoters
30
ENCODE K562 nuclear A- (+ strand)
1
SINE
LINE
LTR
Simple
C
Total RNA
-1
+1
+2
+3
+4
+5
+ - + - + - + - + - + -
- 6557
- 4361
+6
+7
+8
+9
+10
+11
+ - + - + - + - + - + -
- 6557
- 4361
Genomic DNA
-1
6000 -
5000 -
4000 -

Figure S3

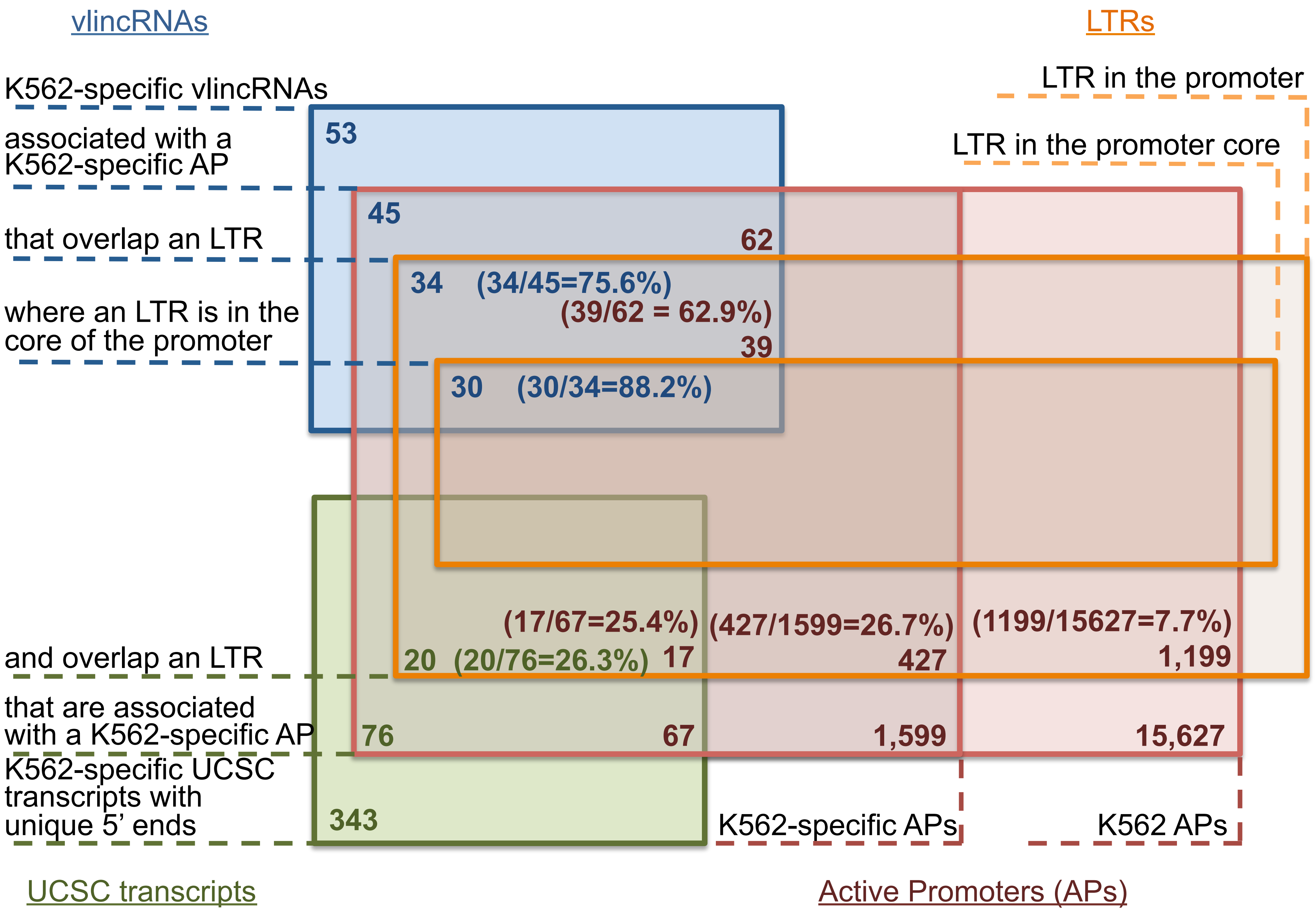

vlincRNAs
LTRs
K562-specific vlincRNAs
associated with a K562-specific AP
that overlap an LTR
where an LTR is in the core of the promoter
LTR in the promoter
LTR in the promoter core
53
45
62
34 (34/45=75.6%)
(39/62 = 62.9%)
39
30 (30/34=88.2%)
(17/67=25.4%)
(427/1599=26.7%)
(1199/15627=7.7%)
and overlap an LTR
20 (20/76=26.3%)
17
427
1,199
that are associated with a K562-specific AP
76
67
1,599
15,627
K562-specific UCSC transcripts with unique 5' ends
343
K562-specific APs
K562 APs
UCSC transcripts
Active Promoters (APs)

Figure S4

EGFP only

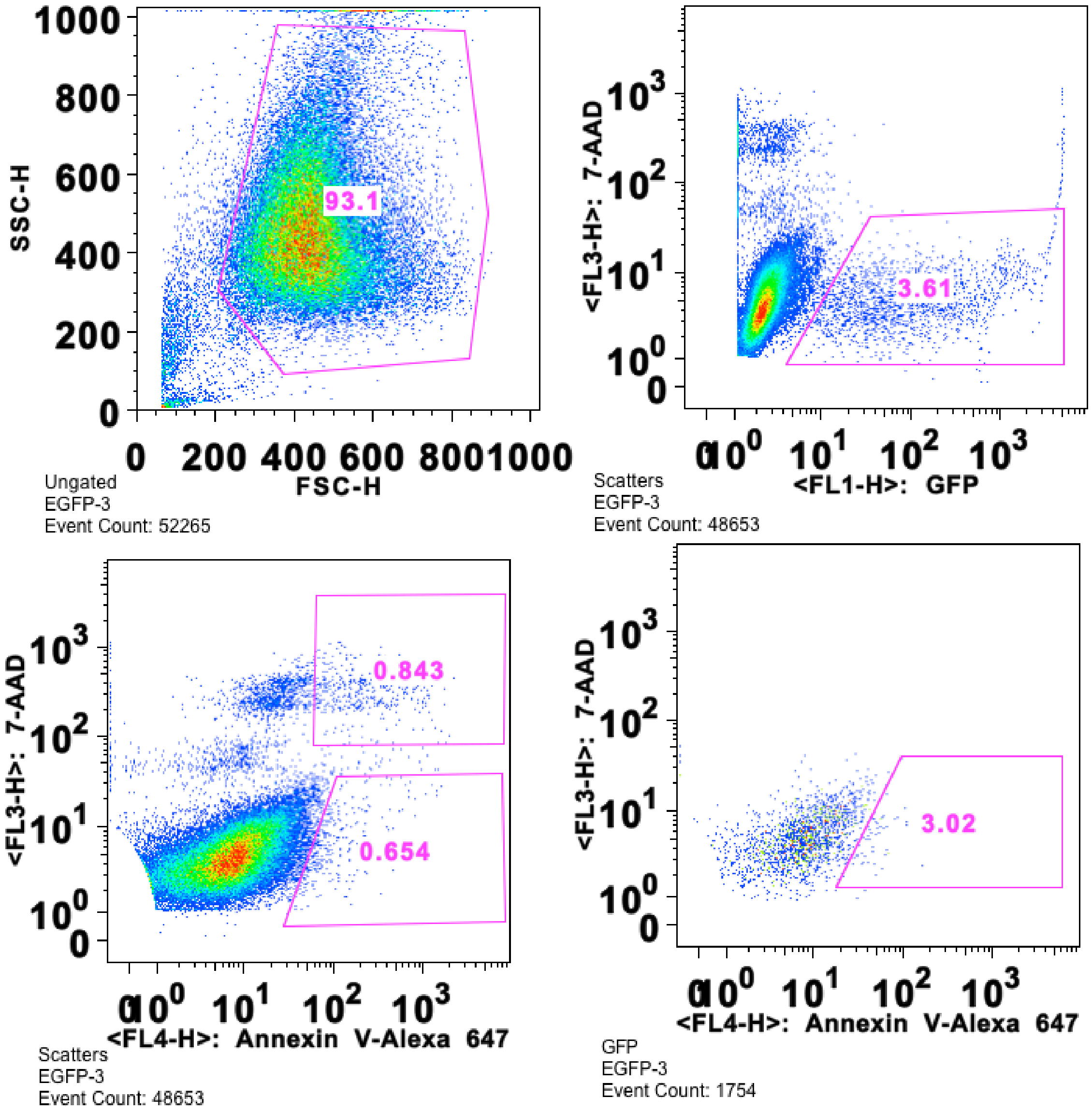

Figure S5

Negative control: Mission siRNA Universal Negative Control #1

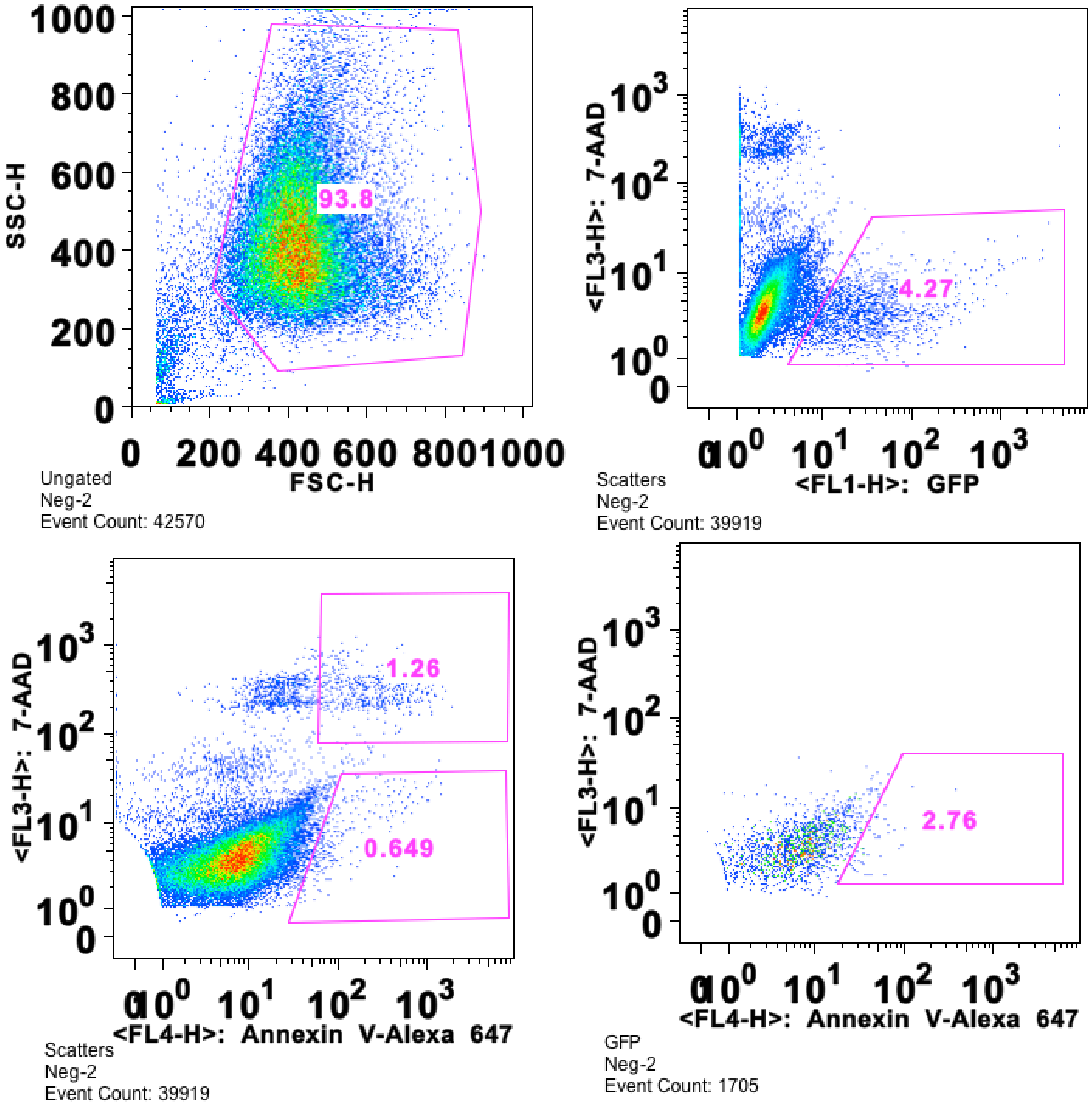

Figure S6

Positive control:AllStars Hs cell Death Control siRNA

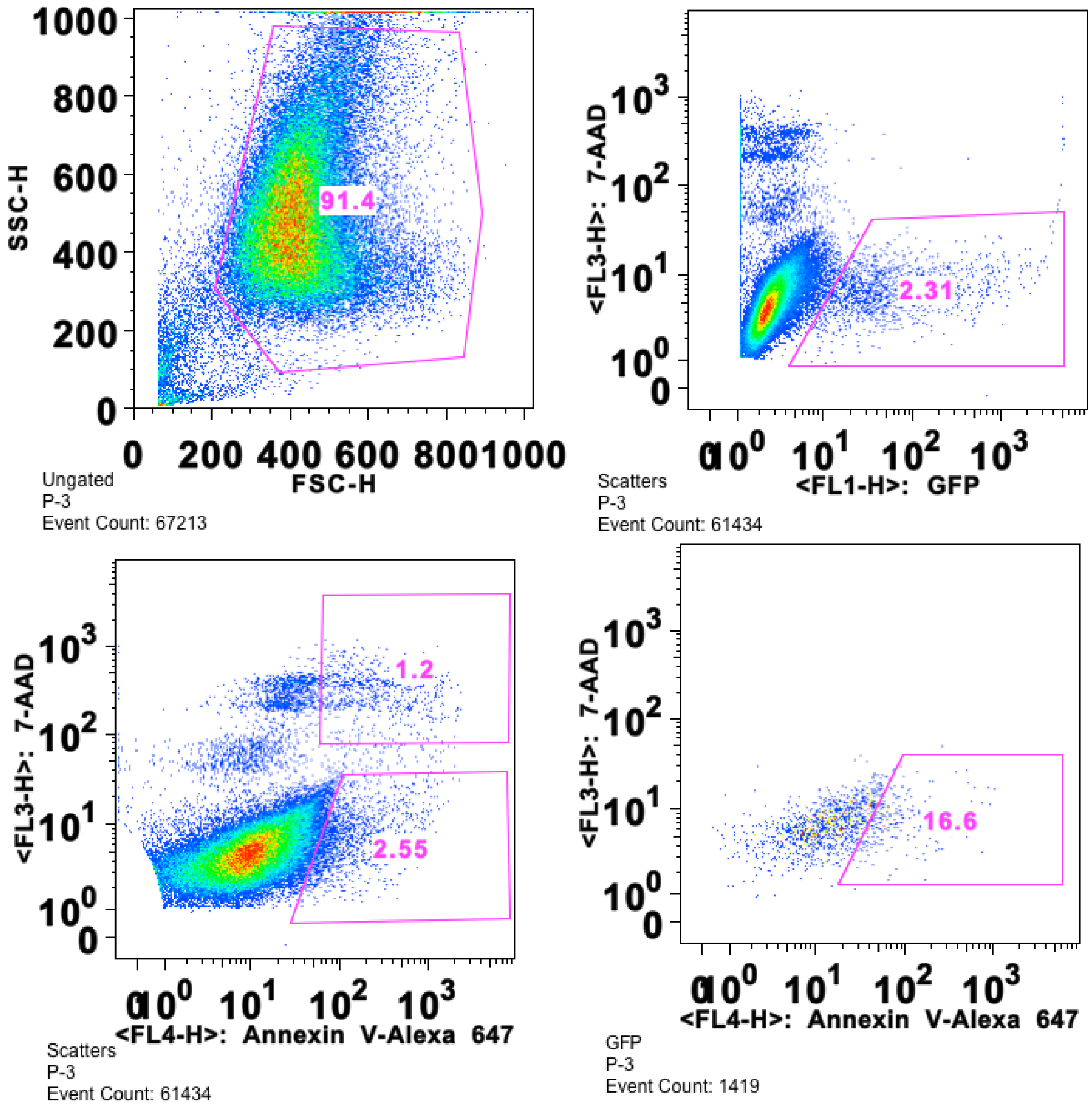

Figure S7

Target siRNA vlinc-21: Negative

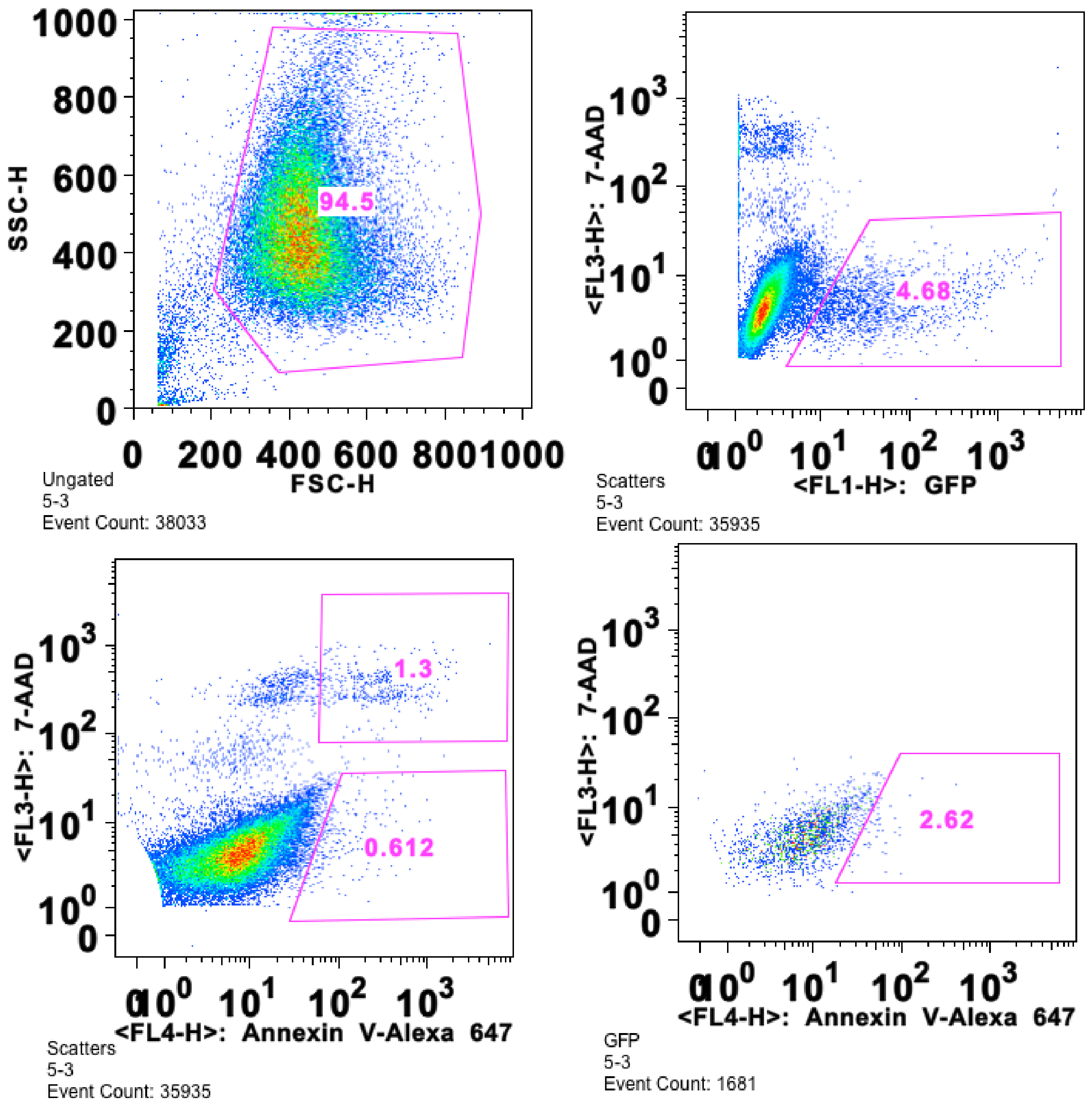

Figure S8

Target siRNA vlinc-243: Positive

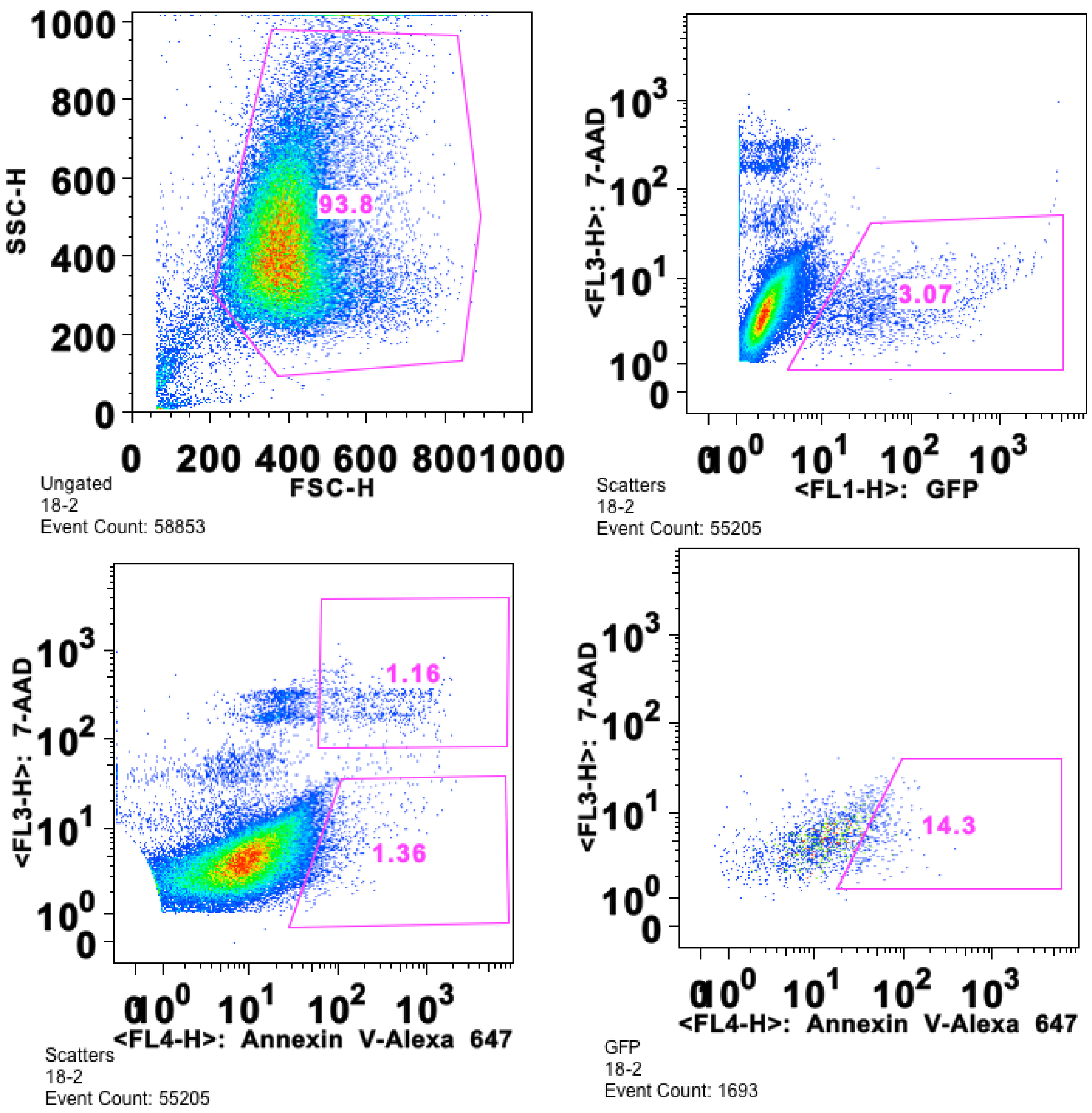

Figure S9
